# Youthful and age‐related matreotypes predict drugs promoting longevity

**DOI:** 10.1111/acel.13441

**Published:** 2021-08-04

**Authors:** Cyril Statzer, Elisabeth Jongsma, Sean X. Liu, Alexander Dakhovnik, Franziska Wandrey, Pavlo Mozharovskyi, Fred Zülli, Collin Y. Ewald

**Affiliations:** ^1^ Department of Health Sciences and Technology Institute of Translational Medicine Eidgenössische Technische Hochschule Zürich Schwerzenbach‐Zürich Switzerland; ^2^ Mibelle Biochemistry, Mibelle AG Buchs Switzerland; ^3^ LTCI Telecom Paris Institut Polytechnique de Paris Palaiseau France

**Keywords:** aging, CMap, collagen, drug repurposing, extracellular matrix, geroprotector, GTEx, longevity, matrisome, pharmacology

## Abstract

The identification and validation of drugs that promote health during aging (“geroprotectors”) are key to the retardation or prevention of chronic age‐related diseases. Here, we found that most of the established pro‐longevity compounds shown to extend lifespan in model organisms also alter extracellular matrix gene expression (*i.e*., matrisome) in human cell lines. To harness this observation, we used age‐stratified human transcriptomes to define the age‐related matreotype, which represents the matrisome gene expression pattern associated with age. Using a “youthful” matreotype, we screened *in silico* for geroprotective drug candidates. To validate drug candidates, we developed a novel tool using prolonged collagen expression as a non‐invasive and *in‐vivo* surrogate marker for *Caenorhabditis elegans* longevity. With this reporter, we were able to eliminate false‐positive drug candidates and determine the appropriate dose for extending the lifespan of *C. elegans*. We improved drug uptake for one of our predicted compounds, genistein, and reconciled previous contradictory reports of its effects on longevity. We identified and validated new compounds, tretinoin, chondroitin sulfate, and hyaluronic acid, for their ability to restore age‐related decline of collagen homeostasis and increase lifespan. Thus, our innovative drug screening approach—employing extracellular matrix homeostasis—facilitates the discovery of pharmacological interventions promoting healthy aging.

## Graphical abstract

We correlated extracellular matrix gene expression signatures (matreotypes) corresponding to young and old human tissues with drug‐treated expression profiles to predict longevity drugs. Then, we established *Caenorhabditis elegans* collagen homeostasis as a novel surrogate marker of longevity as the first pass for *in‐vivo* screening. We validated candidates via lifespan assays and identified new geroprotective drugs.

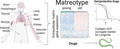

## 1 INTRODUCTION

The demographic shift in the human population reflects an aging society—over 20% of Europeans are predicted to be 65 or over by the year 2025 (Riera & Dillin, 2015). Aging is the major risk factor for developing chronic diseases, such as cancer, Alzheimer's disease, and cardiovascular complications (Partridge et al., 2018). Unfortunately, humans spend on average one‐fifth of their lifetime in poor health suffering from one or multiple age‐related chronic diseases (Partridge et al., 2018). However, the onset of age‐related pathologies is not fixed, and the rate of aging was shown to be malleable. The goal of biomedical research on aging or geroscience is to identify interventions that compress late‐life morbidity to increase the period spent healthy and free from disease.

A few geroprotective drugs exist that postpone age‐related diseases (Riera & Dillin, 2015). For instance, the anti‐diabetes drug metformin reduces age‐related chronic diseases and mortality from all causes (Bannister et al., 2014). Ongoing clinical trials on geroprotective drugs or compounds include the anti‐diabetic drugs metformin (NCT02432287, NCT03451006) and acarbose (NCT02953093); mTOR‐inhibiting and immunosuppressant drug rapamycin (sirolimus; NCT02874924); natural compounds resveratrol (NCT01842399) and urolithin A (NCT04160312); and nicotinamide adenine dinucleotide precursors NR (NCT02950441) and NMN (NCT04685096). One primary outcome measure used in the aforementioned clinical trials for metformin and acarbose is the restoration from an “old” to a “youthful” gene expression signature (NCT02432287, NCT02953093). Therefore, we reasoned that cross‐comparing youthful expression signatures against expression profiles elicited by small molecules could identify geroprotective compounds. Conceptually similar *in* *‐* *silico* approaches have been conducted in the past (Aliper et al., 2016; Calvert et al., 2016; Dönertaş et al., ,^2018^, ^2019^; Fuentealba et al., 2019; Janssens et al., 2019; Komljenovic et al., 2019; Liu et al., 2016). However, these former approaches comparing age‐related expression profiles with drug‐treated cells revealed similar drug targets (Dönertaş et al., 2019), such as the HSP90/HSF‐1 axis (Fuentealba et al., 2019; Janssens et al., 2019). Our strategy was to use a more refined starting list of a “youthful” gene expression signature with experimentally implicated genes associated with healthy aging.

A key signature of aging is the continuous decline of collagen and cell adhesion gene expression (Ewald, 2019; Magalhães et al., 2009) accompanied with an increase in matrix metalloproteinase expression (Ewald, 2019). Gene expression ontologies of extracellular matrix (ECM) genes have been associated with healthy aging in humans (Zeng et al., 2020). The ECM not only embeds cells and tissues but also provides instructive cues that change cellular function and identity. For instance, placing old cells into a “young” ECM rejuvenates senescent cells or stem cells and even reprograms tumor cells (reviewed in (Ewald, 2019)). Moreover, collagen homeostasis is required and sufficient for longevity in *Caenorhabditis elegans* (Ewald et al., 2015). Heparan/chondroitin biosynthesis and TGFβ pathway are frequently enriched in *C. elegans* longevity drug screens (Liu et al., 2016). Collectively, these functionally implicated genes are all members of the matrisome.

The human matrisome encompasses 1027 protein‐encoding genes that either form the ECM, such as collagens, glycoproteins, and proteoglycans; associate with ECM (*e.g*., TGFβ, Wnts, and cytokines); or remodel the ECM (*e.g*., matrix metalloproteinases (MMPs)) (Naba et al., 2016). The matrisome represents about 4% of the human genome and is functionally implicated in about 8% of the total 7037 unique human phenotypes (Statzer & Ewald, 2020). Age‐related phenotypes rank among the top matrisome‐phenotypic categories across species (Ewald, 2019; Taha & Naba, 2019). Proteomics approaches have revealed unique ECM compositions associated with health and disease status (Socovich & Naba, 2019). ECM compositions can even be used to identify distinct cancer‐cell types (Ewald, 2019). Therefore, organismal phenotypes, physiological states, and cellular identity are characterized by distinct sets of expressed ECM proteins. Since these unique ECM compositions are an expression profile on a temporary, sometimes local basis and do not involve the entire matrisome, we coined the term matreotype (Ewald, 2019). The matreotype is the acute state of an ECM composition associated with—or causal for—a given physiological condition or phenotype (Ewald, 2019).

Given the functional implication of ECM in healthy aging, we hypothesized that a youthful matreotype might predict drugs promoting healthy aging. Here, we define a youthful human matreotype using data from the Genotype‐Tissue Expression (GTEx) project (Consortium, 2013). We query this young matreotype signature with the drug resource Connectivity Map (CMap) (Lamb et al., 2006) data to identify longevity‐promoting compounds. We then developed a novel *in‐vivo* tool as a surrogate marker for longevity to find appropriate drug doses to be used for *C. elegans*' lifespan assays. Our results implicate previously known longevity drugs as well as novel drugs, providing a proof‐of‐concept for our approach.

## 2 RESULTS

### 2.1 Geroprotective compounds associated with altering ECM

**FIGURE 1:**
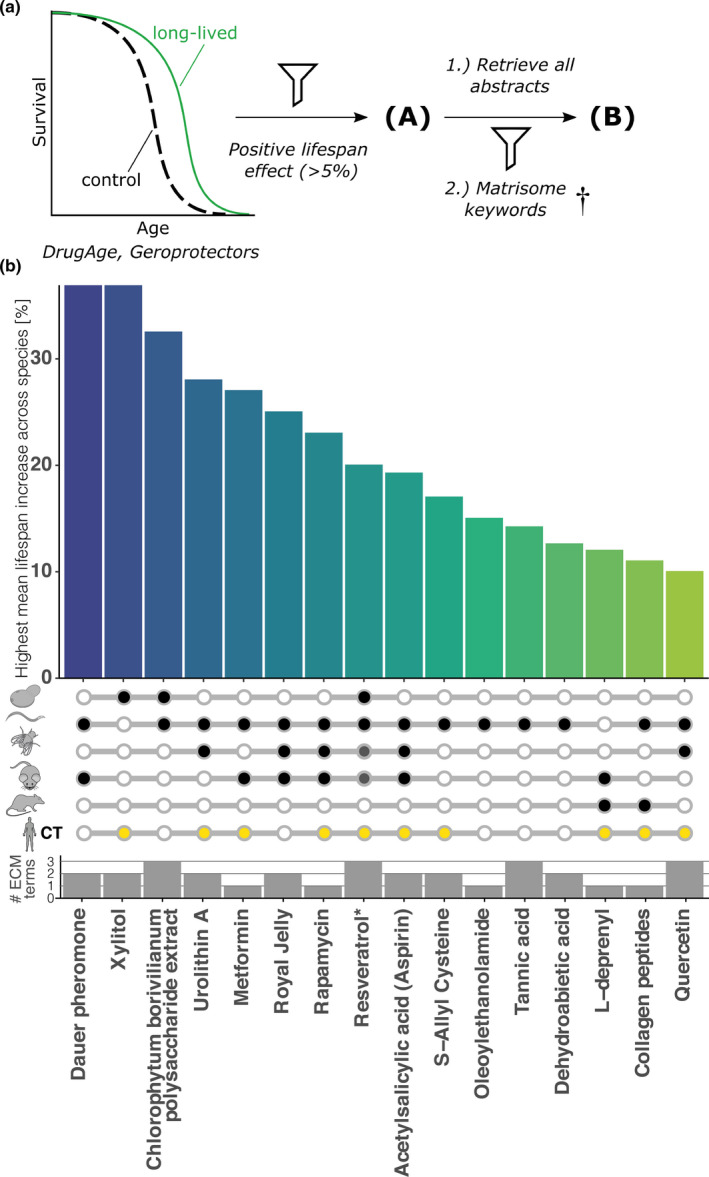
Geroprotective compounds shown to affect the ECM. (a) Schematic depiction of the literature and database mining approach used. (b) Compounds which contained ECM keywords in their abstract were ranked by their highest mean lifespan increase in any species. Open circle = not assessed, black closed circle = lifespan increased, and yellow circle = compound assessed in clinical trials (CT). The number of ECM keywords quantified in the abstract is displayed below. *Resveratrol only on a high‐fat diet increased lifespan in fly and mouse. For details and references, see Table S1

We first performed literature and database mining to search for compounds that have been shown to increase lifespan and are known to alter ECM in any organism. We acquired lifespan data from databases DrugAge and GeroProtectors (Figure 1a) (Barardo et al., 2017; Moskalev et al., 2015). We filtered for reported mean lifespan extensions that were above 5% for compounds compared to control (Figure 1a; Table S1)—then queried all PubMed abstracts for the given agent and our ECM key terms, including collagen, MMP, proteoglycan, integrin, and TGFβ (Figure 1a; Table S1). After manual curation, we identified 3% (16 out of 567) of the examined known longevity‐promoting compounds that slow aging and also had been reported to affect proteins outside of cells, such as collagens and other matrisome proteins (Figure 1b; Table S1).

### 2.2 Longevity compounds affect matrisome gene expression

**FIGURE 2:**
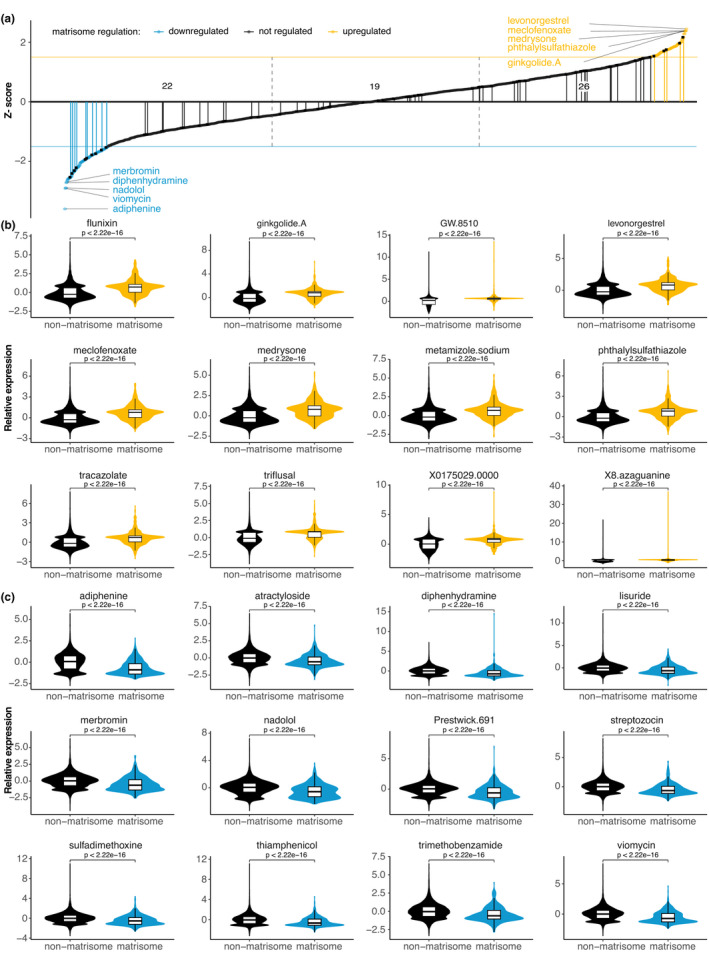
Drugs affecting matrisome gene expression. (a) Z‐score of altering matrisome gene expression across all 1309 CMap drugs. Vertical lines indicate compounds shown to increase lifespan in any organism. (b–c) Top 12 compounds that increase (b) or decrease (c) the overall matrisome gene expression. For details, see Table S2

Next, we investigated whether compound treatments, in general, would alter matrisome expression. Connectivity Map (CMap) is a library of 1.5 million gene expression profiles comparing 1309 different compound treatments on human cell cultures (Lamb et al., 2006). We queried the CMap library for compound treatments that either increase or decrease the expression levels of matrisome genes. Using a z‐score threshold of ±1.5, we identified 167 compounds that strongly regulate the 594 out of the 1027 matrisome genes compared to the background (13,752 total quantified genes; Figure 2a, Figure S1, Table S2). Out of the 12 most up‐ and 12 most downregulated matrisome expression profiles upon a compound treatment, we identified ten agents linked to longevity or impairment of age‐related pathologies (Figure 2b,c, Table S2). Strikingly, out of these 47 known longevity‐promoting compounds that were assessed in the CMap library, we found 41 (87%) significantly altered matrisome gene expression (Figures [Link], [Link], [Link], Table S2). After manual curation, we identified 20 additional compounds reported to increase lifespan. From the total 67 reported lifespan‐increasing compounds, 19 had minor effects on overall matrisome gene expression, whereas 26 compounds increased, and 22 decreased, matrisome gene expression (Figure 2a, Table S2). These results suggest that compounds implicated in healthy aging show enriched differential matrisome gene expression.

### 2.3 Defining young and old human matreotypes

For a more targeted approach, we reasoned that using a young age‐associated matrisome gene expression signature (*i.e*., youthful matreotype) to query CMap data should reveal more compound treatments that might promote longevity. To define a young and old human matreotype, we built upon the previous analysis of the GTEx dataset (Consortium, 2013) comparing a young versus an old gene expression pattern of more than 50 tissues and 8000 transcriptomes by Janssens and colleagues (Janssens et al., 2019). From these 50 tissues, we only identified 15 tissues that had on average 138 age‐related transcripts per tissue, which we then filtered for matrisome genes (Figures S5, S6, Table S3). In our analysis, we used two different approaches to quantify age‐related transcript changes: the difference in expression (“absolute”) and the fraction of change (“relative”). The absolute expression change preferentially captures genes exhibiting high baseline expression levels, since any change in their abundance translates to a large absolute change. By contrast, the relative expression difference quantifies the change to the previously measured value and favors lower expressed genes.

**FIGURE 3:**
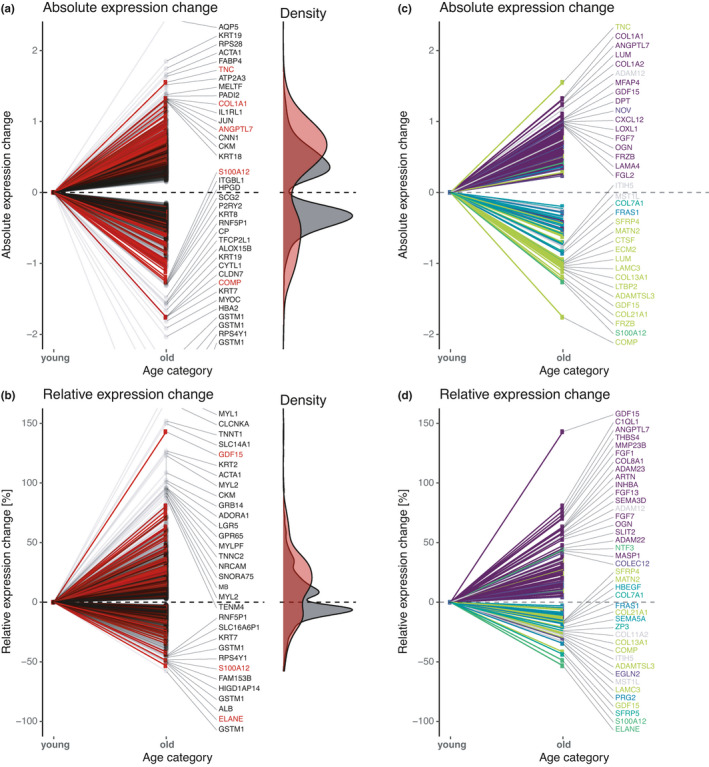
Matrisome genes affected by aging across human tissues. (a–b) Absolute (a) and relative (b) expression changes of all detected genes during aging are shown. Matrisome genes are displayed in red and non‐matrisome genes in black. (c–d) Absolute (c) and relative (d) expression changes of only detected matrisome genes during aging are shown and are colored by tissue. For details, see Table S3

Among these age‐related transcripts, matrisome genes were well‐represented: collagen gene expression decreased with age, while matrix proteases (MMPs, ADAMs) increased (Figure 3a–d, Table S3). These findings were consistent with previous reports (Ewald, 2019; Magalhães et al., 2009). However, we noted a number of key observations. First, each tissue has a unique age‐related matreotype gene expression signature (Figures S5, S6, Table S3). Second, both the transcript coverage and the age association of the matrisome varies within the 15 assessed tissues. Therefore, we decided to narrow the focus of our study to five tissues: skin, thyroid, pituitary, aorta, and coronary artery tissue (Figure S7, Table S3). In the remaining tissues, the number of age‐associated transcripts was too low to quantify the contribution of the matrisome conclusively. Third, certain matrisome genes, such as GDF15, experienced both increasing and decreasing expression levels during aging, depending on the tissue (Figure 3b). With these observations in mind, our aim was to construct a multi‐tissue compendium of matrisome members, which were most affected by aging.

To extend the matreotype generated from these five tissues, we combined the findings of eight studies that also included transcriptomes across ages from different tissues in order to validate and identify additional multi‐tissue and age‐related matrisome genes (Table S3). By considering both absolute and relative aging gene expression changes, we determined the age‐related matreotype of brain, fat, skin, and other tissues to generate a new common signature across tissues (Figure S8, Table S3). By inverting the aged expression pattern, we defined the age‐reversed or “youthful” matreotype (Table S4). Using this approach, we defined here, for the first time, a multi‐tissue compendium of 99 genes across 15 human tissues, which we define as the young and aged matreotype.

### 2.4 Use of the young and aged matreotype to identify new pro‐longevity compounds

**FIGURE 4:**
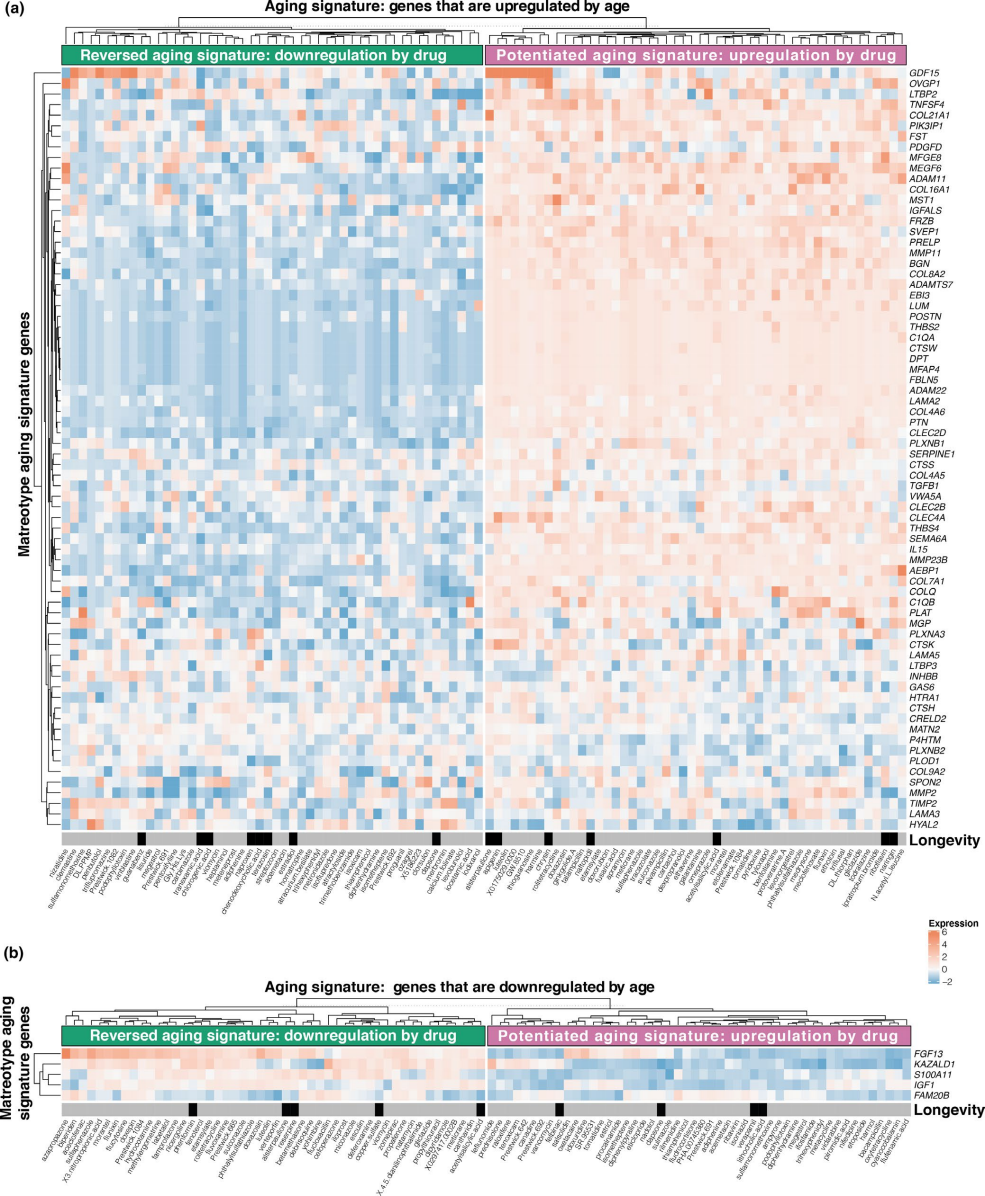
Matreotype signature gene expression correlating with CMap expression pattern. (a–b) Matreotype genes that are strongly upregulated (a) or downregulated (b) during aging are hierarchically clustered with gene expression patterns of small molecules on human cell lines from CMap library (Table S4). Black rectangles indicate that the respective compound increases lifespan in any organism (Table S5)

The ultimate goal of our matreotype signature is its application to identify new geroprotective compounds that modulate the matrisome, and thus, longevity. To first validate this approach, we used known pro‐longevity compounds and identified those causing a youthful matreotype (*i.e.,* similar gene expression; Figure 4, Tables S4, S5). We parsed the youthful matreotype signature into “downregulated matreotype genes that become upregulated during aging” (Figure 4a) and “upregulated matreotype genes that become downregulated during aging” (Figure 4b, Table S4). We queried the CMap compound expression profiles and plotted the top 50 compounds that showed the gene expression signature as predicted by the youthful matreotype and called them “reversed aging signature” compounds (Figure 4a,b). As a control, we also plotted the top 50 compounds that would enhance gene expression in the directionality of aging and called them “potentiated aging signature” compounds (Figure 4a,b). Of these total 200 compounds, 15 compounds were identified with both youthful and aged matreotypes, leaving 185 unique compounds.

Next, we searched the literature for reported lifespan increase in any organism upon treatment with any of these 185 compounds and found 24 of these compounds resulted in lifespan extension (Figure 4, Table S5). To our surprise, these 24 compounds with reported lifespan increase did not cluster preferentially with the “reversed aging signature” compounds as predicted but rather were almost equally distributed among all categories (Figure 4). This suggests that at least for matrisome genes, longevity might not be a simple reversion of gene expression associated with aging. Or it might be more complex given that each tissue has a unique matreotype (Figures [Link], [Link], [Link]), such as that seen with GDF15 (Figure 3). Despite this unexpected finding, we note that our matreotype‐99‐gene‐compendium was able to identify 185 unique compounds, of which 13% have previously been reported to extend lifespan (Figure 4). This is an improvement compared to the 5% of the 67 longevity‐promoting compounds found in all 1309 CMap assessed small molecules (Figure 2a). Thus, independent of directionality, both youthful and age‐related matreotype, that is, the matreotype signature itself predicts longevity‐promoting drugs.

### 2.5 Validating matreotype‐predicted compounds with lifespan assays using *C. elegans*

**FIGURE 5:**
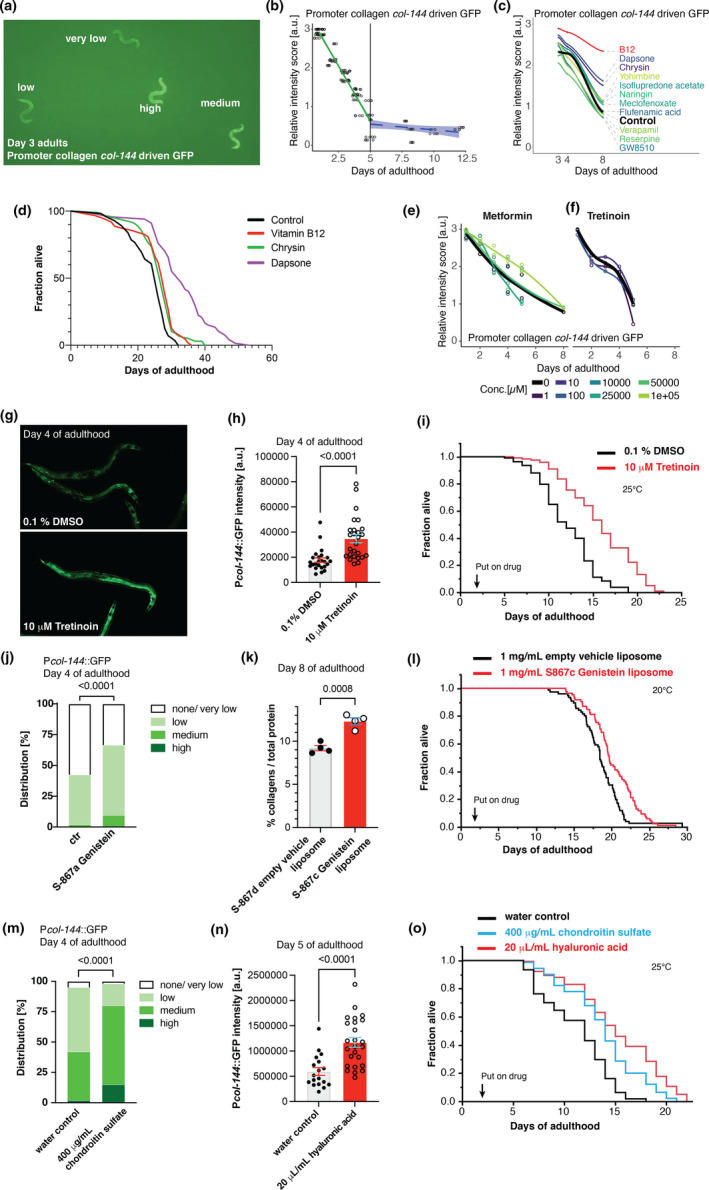
Validating compounds that were predicted to modulate the age‐related matreotype for *in‐vivo* collagen expression and lifespan. (a) Heterogeneity in decline of collagen *col*‐*144* promoter‐driven GFP (LSD2002 [P*col*‐*144*::GFP]) at day 3 of adulthood. GFP levels were categorized in 4 groups (none/very low, low, medium, and high). (b) Approximately linear decline of P*col*‐*144*::GFP expression during the first 5 days of adulthood. (c) A single concentration per matreotype‐predicted compound tested for their efficacy on prolonging P*col‐144*::GFP expression during aging. (d) Lifespan corresponding to the matreotype‐predicted compounds with prolonged P*col‐144*::GFP expression during aging. (e–f) Metformin (e) and tretinoin (f) were observed to partially prolong P*col*‐*144*::GFP expression during aging in certain dosage regimes. (g–h) Representative image (g) and quantification (h) of P*col*‐*144*::GFP expression at day 4 of adulthood upon tretinoin treatment from day 1 of adulthood. (i) Adulthood treatment (start at day 2 of adulthood) of tretinoin extended lifespan. (j) Adulthood treatment of genistein increased P*col*‐*144*::GFP expression at day 4 of adulthood. (k) Liposomal‐encapsulated genistein showed higher collagen over total protein levels at day 8 of adulthood. (l) Adulthood treatment (start at day 2 of adulthood) of liposomal‐encapsulated genistein extended lifespan. (m) Adulthood treatment of chondroitin increased P*col*‐*144*::GFP expression at day 4 of adulthood. (n) Adulthood treatment of hyaluronic acid increased P*col*‐*144*::GFP expression at day 5 of adulthood. (o) Adulthood treatment (start at day 2 of adulthood) of chondroitin or hyaluronic acid extended lifespan. For raw data and statistics for (b, c, e, f, h, j, k, m, n), see Table S6. For raw data, statistics, and additional trials (d, i, l, o), see Table S7

To translate the *in* *‐* *silico* analysis to an *in‐vivo* functional relevance for healthy aging, lifespan assays in model organisms, such as *C. elegans* or mice, are commonly used. The limitation of these lifelong assays, especially in mice, is that often one does not know if the optimal dose is applied until the end of the study 3 years later. To overcome this limitation, we developed an *in‐vivo* screening assay measuring collagen biosynthesis. Similar to humans, collagen biosynthesis declines with age in *C. elegans*, and we recently discovered that many, if not all, longevity interventions prolong the expression of collagen genes in *C. elegans* (Ewald et al., 2015). This prolonged expression of key collagen genes is required and sufficient for longevity (Ewald et al., 2015). Thus, we hypothesized that prolonged collagen expression, quantified on the transcriptional level by a collagen promoter‐driven GFP, would constitute a useful surrogate marker to predict longevity (Figure 5a). Indeed, we found that the GFP intensity driven by collagen *col*‐*144* promoter (P*col‐144*::GFP) declined almost linearly within the first 5 days of adulthood (Figure 5b, Table S6).

To validate our hits, we chose eleven compounds out of 194 matreotype‐predicted hits and examined a single compound dose for examining the P*col‐144*::GFP expression levels during aging. We found that vitamin B12 (cyanocobalamin), chrysin, and dapsone maintained strong P*col‐144*::GFP expression at old age (Figure 5c, Table S6). Therefore, we decided to assess these concentrations of B12, chrysin, and dapsone, and found that they robustly increased lifespan (Figure 5d, Table S7). This indicates the usefulness of this reporter to identify a longevity‐promoting dose for *C. elegans* lifespan validation.

In our youthful matreotype‐associated drugs, we have identified phenformin (Figure 4b, Figure S9, Table S2), an analog of metformin. Both phenformin and metformin have been shown to increase *C. elegans* lifespan (Pryor & Cabreiro, 2015). Here, we found that treating late L4 *C. elegans* with metformin, prolonged collagen expression in a dose‐dependent manner (Figure 5e; Figure S10, Table S6). This is consistent with a study showing metformin slows extracellular matrix morphological decline of the cuticle (Haes et al., 2014). This suggests that one unexplored aspect of the metformin's mechanism of action might be via improved collagen homeostasis. Given this exciting finding, we decided to prioritize our investigations into drugs that will enhance collagen homeostasis in mammals but have not shown any pro‐longevity phenotypes in any organisms. We, therefore, chose to test the retinoic acid receptor agonist tretinoin since tretinoin treatment prevents MMP upregulation and stimulates collagen synthesis in photo‐aged skin (Griffiths et al., 1993; Mukherjee et al., 2006). Our analysis showed that tretinoin had an enriched differential expression of matrisome genes (Figure S9). Furthermore, tretinoin has been predicted to associate with a youthful expression pattern by the *in* *‐* *silico* analysis of Janssens and colleagues but did not increase *C. elegans* lifespan at 50 μM (Janssens et al., 2019). With our reporter system, we found that treatment with 10 μM of tretinoin prolonged collagen expression and increased lifespan (Figure 5f–i; Figure S10, Tables S6, S7), confirming that tretinoin indeed promotes healthy aging.

Following the same rationale, in our drug hits that change matrisome expression, we identified genistein, an isoflavone (phytoestrogen) derived from soybeans to have a youthful matreotype profile (Figure S9). Genistein has been predicted to associate with a youthful expression pattern by a previous *in* *‐* *silico* analysis but failed to increase *C. elegans* lifespan at 50 μM (Janssens et al., 2019). By contrast, other groups reported a lifespan increase using 50 and 100 μM genistein (Lee et al., 2015). To reconcile this, we generated new 98% pure genistein and found prolonged collagen expression in aged *C. elegans* and a mild increase in lifespan (Figure 5j, Figure S11, Table S7). To optimize drug uptake, we encapsulated genistein extracts with liposomes. We found higher collagen protein content in aged *C. elegans* (day 8 of adulthood), enhanced oxidative stress resistance during older age (day 5 adults), and improved lifespan extension compared to empty vector liposomal control treatments (Figure 5k,l, Figures [Link], [Link], Tables S7–S8). A similar strategy was used to encapsulate royal jelly oil in a lecithin‐based nanoemulsion for improved solubility on the NGM culturing plates to facilitate uptake by *C. elegans* (Figures S10, S11). These data indicate that optimizing drug delivery and dose is important for lifespan and healthspan benefits.

To expand our findings, we searched for compounds that could alter ECM composition and were not included in the CMap library. Besides collagens, glycoprotein and proteoglycans are the other two major components of ECMs across species (Naba et al., 2016; Teuscher et al., 2019). We decided to investigate ECM precursors, such as glucosamine, chondroitin sulfate, and hyaluronic acid, which are part of the sugars added to these ECM proteins as potential mediators of the youthful matreotype. In humans, cohort studies of over 70–500 thousand participants who took glucosamine or chondroitin supplements showed a 15%–18% and 22% reduction in total mortality, respectively (Bell et al., 2012; Li et al., 2020). Here, we found that glucosamine treatment increased collagen expression during aging (Figure S10, Table S6). Previous studies also implicated glucosamine supplementation in lifespan extension of *C. elegans* and mice (Weimer et al., 2014). Using our collagen reporter screening system, we determined that 20 μl/ml of hyaluronic acid and 400 μg/ml chondroitin sulfate prolonged collagen synthesis during aging and were sufficient to increase lifespan (Figure 5m–o; Figures S10, S12, Tables S6–S8). Thus, our ECM‐transcriptional reporter system is a powerful tool to identify drug‐response doses that promote healthy aging.

## 3 DISCUSSION

Recent artificial intelligence, *in silico*, and other computational approaches have been harnessed to predict beneficial and longevity‐promoting effects of compounds, which were previously not considered to mediate effects on ECM gene expression. A major challenge lies in the validation of the health‐promoting results of a compound. Here, we demonstrated that a concise list of 1027 matrisome or 99 matreotype genes facilitates the identification of lifespan‐enhancing drugs. To establish a proof‐of‐concept for our matreotype approach, we developed a new non‐invasive and *in* *‐* *vivo* reporter system, which we used to validate known and novel geroprotective drugs. We used our system to determine the appropriate dose to unravel the compounds' longevity potential indicated with our, and previous, *in* *‐* *silico* approaches.

Established geroprotective drugs, such as metformin, rapamycin, resveratrol, and others, with known lifespan‐extending effects, have previously been reported to alter the expression of ECM components (Figure 1). When we compared gene expression signatures, we found that almost ninety percent of the known longevity‐promoting compounds in the CMap library showed changes in matrisome gene expression. To identify novel geroprotective drugs, we refined our approach by parsing ECM gene signatures resembling a young or aged matreotype to correlate with a given drug's gene expression pattern. We found 185 candidate drugs, of which 24 showed lifespan increase in model organisms and 42 unique compounds had previously been predicted as potential geroprotectors (Table S5).

The generally accepted assumption is that there is a drift in gene expression during aging, and that restoration of a younger gene expression pattern indicates rejuvenation of cells or tissues. This premise has been extensively used as a biomarker for the restoration of health in clinical trials (NCT02432287, NCT02953093), parsing healthy versus common aging cohorts (Zeng et al., 2020), reprogramming cells into a younger state (Lu et al., 2020), or in previous *in* *‐* *silico* approaches (Tyshkovskiy et al., 2019). This premise also requires that longevity or rejuvenating interventions work through temporal scaling, a process that has been shown to be the case for lifespan extension and aging‐associated gene expression in *C. elegans* (Stroustrup et al., 2016; Tarkhov et al., 2019) but not yet for mammals. We found that longevity‐promoting drugs either increase or decrease matrisome gene expression (Figure 2). With a more refined approach using the youthful matreotype, we observed that both reversing or propagating the aging gene signatures could predict geroprotective drugs (Figure 4). One explanation could be that there is overlap in the gene expression signatures during aging and chronic diseases are similar (Zeng et al., 2020).

Clearly, independent of directionality, the current defined age‐related matreotype holds predictive power to identify new lifespan‐enhancing drugs. A shortcoming of our definition of the aging‐ and youth‐associated expression signatures is that we do not take the context of the individual tissues into consideration. Unfortunately, insufficient studies are available to compile high‐quality subsets for each tissue, which should be addressed in further experimental investigations. This is especially important in the case of collagen expression at an advanced age that can be both associated with improved tissue maintenance as observed in the skin and joints, while at the same time be implicated in fibrotic changes in the liver and kidney. For instance, downregulation of ECM in fat tissue but not in blood vessels is a key gene signature for healthy elderly individuals (Zeng et al., 2020). During aging, inflammation increases leading to fibrosis (Ewald, 2019). On the contrary, collagen synthesis declines during aging and degradation or fragmentation by increased MMP activities, evident in aging skin (Ewald, 2019). Several drugs, including rapamycin (Chung et al., 2019), tretinoin (Griffiths et al., 1993; Mukherjee et al., 2006), genistein (Polito et al., 2012), and resveratrol (Lephart & Andrus, 2017), increase collagen synthesis in the skin. By contrast, resveratrol, rapamycin, and genistein suppress fibrotic collagen deposition by intestinal fibroblasts, kidney, and lung tissues, respectively (Chen et al., 2012; Li et al., 2014; Matori et al., 2012). Thus, drugs might act as a geroprotector and increase lifespan by either inhibiting or enhancing the matrisome expression depending on pre‐existing tissue damage or disease.

The model organism *C. elegans* does not show inflammation nor fibrosis during aging. Thus, collagen expression in *C. elegans* might reflect restoration or repair of the progressive decline of ECM homeostasis during aging analogous to human skin. This makes *C. elegans* the ideal readout for any age‐dependent changes of matrisome genes observed from human or mammalian omics approaches. Based on this, we established an age‐dependent collagen transcriptional read‐out as a predictive marker for longevity.

In previous work, more than 100,000 compounds have been screened, and about 100 compounds have been identified to increase *C. elegans* lifespan (Table S5). A practical limitation in verifying drug candidates is the uncertainty in optimal dosage levels. Usually, one dose is chosen for all compound treatments for *C. elegans* lifespan screening assays, potentially leading to many false‐negative results and limiting its interpretation. Dose‐response curves are not linear, but often J‐ or U‐shaped, whereby in general, high doses are toxic and low doses lead to hormetic responses increasing lifespan. We showed that two previously predicted but regarded as false‐positive compounds—tretinoin and genistein—robustly increase lifespan when assayed at the appropriate dosage. Furthermore, optimization for the route of uptake improves robustness, promoting healthy aging. Thus, our reporter system serves as a predictive tool to identify the appropriate dosage for lifespan assays.

It is striking that longevity‐promoting compounds identified in model organisms showed enriched matrisome gene expression signature in human cells treated with these compounds (Figures [Link], [Link], [Link]). The proposed mechanisms for longevity compounds discovered with *C. elegans* mostly work through intercellular communication. Pathway analysis showed an enrichment for chondroitin and heparan sulfate biogenesis and TGFβ pathway as predicted drug‐protein targets (Liu et al., 2016). On the contrary, the outcome of previous *in* *‐* *silico* approaches using youthful gene expression determined from GTEx data to query CMap identified HSP90 chaperone network (Dönertaş et al., 2019; Fuentealba et al., 2019; Janssens et al., 2019) and protein homeostasis (Komljenovic et al., 2019) as healthy aging promoting interventions. HSP90 is found in the extracellular space binding fibronectin and chaperones other ECM proteins (Hunter et al., 2014). Pharmacological inhibition of HSP90 alters ECM signaling (Chaturvedi et al., 2011). Proper ECM protein homeostasis is essential to ensure intercellular communication, a hallmark lost during aging (Ewald, 2019). This raises the question whether geroprotective drugs improve ECM homeostasis. There is tantalizing evidence with established longevity‐promoting medications, such as rapamycin, resveratrol, metformin, and others (Figure 1b), and we provided experimental evidence for this with tretinoin, genistein, glucosamine, chondroitin, and hyaluronic acid (Figure 5). Consistent with this is that glucosamine and chondroitin stimulate collagen synthesis *in vitro* and *ex vivo* of elderly human skin in a clinical trial (Gueniche & Castiel‐Higounenc, 2017; Lippiello, 2007), extracellular matrix component hyaluronic acid treatments stimulate collagen synthesis in human photo‐aged skin (Wang et al., 2007), and in mice (Fan et al., 2019), and topical application of rapamycin restores collagen VII levels in a clinical trial (NCT03103893) (Chung et al., 2019). ECM homeostasis might remodel or prevent glycation and crosslinking of collagens (Ewald, 2019). There are currently 27 clinical trials addressing ECM stiffness and its role in diseases by investigating eleven different molecular targets. Furthermore, different matreotypes might be valuable prognostic factors or biomarkers (Ewald, 2019). Thus, defining matreotypes related to healthy aging or age‐related chronic diseases might be a strategy for personalized medicine approaches.

In summary, we demonstrated that prolonged ECM expression is a biomarker for *C. elegans* longevity and harnessed this to establish a novel *in* *‐* *vivo* assay. We provided evidence that gene expression patterns of human cells treated with known geroprotective drugs alter ECM genes and developed an age‐stratified matreotype. We then used this matreotype to identify geroprotective compounds based on their transcriptomes. Our method highlights a previously unused potential of ECM reprogramming as a means to identify and validate compounds, licensed drugs, natural compounds, and supplements that potentially retard or prevent age‐related pathologies. Understanding pharmacological reprogramming of extracellular environments may provide a new platform to discover previously unidentified therapeutic avenues and holds significant translational value for disease diagnostics.

## 4 EXPERIMENTAL PROCEDURE

### 4.1 Strains

*Caenorhabditis elegans* strains were maintained on NGM plates and OP50 *Escherichia coli* bacteria. The wild‐type strain was N2 Bristol. Mutant strains used are described at www.wormbase.org: TJ1060: *spe*‐*9(hc88)* I; *rrf*‐*3(b26)* II. LSD2002 was generated by integrating P*col*‐*144*::GFP transgene (a generous gift from Yelena Budovskaya and Stuart Kim) into N2, outcrossing eight times, and crossing to TJ1060, resulting *spe*‐*9(hc88)* I; *xchIs001* [P*col*‐*144*:: GFP; *pha*‐*1*(+)] X genotype.

### 4.2 Data analysis

Data analysis was performed utilizing the dplyr (Hadley Wickham, Romain François, Lionel Henry and Kirill Müller (2020). dplyr: A Grammar of Data Manipulation. R package version 1.0.0) and purrr (Lionel Henry and Hadley Wickham (2020). purrr: Functional Programming Tools. R package version 0.3.4). R packages. Data visualization was generated using ggplot2 (H. Wickham. ggplot2: Elegant Graphics for Data Analysis. Springer‐Verlag New York, 2016), ComplexHeatmap (Gu (2016). Complex heatmaps reveal patterns and correlations in multidimensional genomic data. Bioinformatics), and ggpubr (Alboukadel Kassambara (2020). ggpubr: “ggplot2” Based Publication Ready Plots. R package version 0.4.0.999).

### 4.3 Data origin

Human matrisome (http://matrisomeproject.mit.edu, (Naba et al., 2016)), GTEx data (Consortium, 2013), age change (Janssens et al., 2019). CMap data (Lamb et al., 2006), lifespan information were obtained from GenAge, Geroprotectors (Moskalev et al., 2015), and through a comprehensive literature review.

### 4.4 Literature search

Compounds that extended the mean lifespan of the organism by more than 5%, according to the DrugAge (Barardo et al., 2017) and Geroprotector (Moskalev et al., 2015) databases, were selected for further investigation. The abstracts of studies associated with lifespan extension were filtered for the occurrence of at least one of the following matrisome keywords: collagen, ECM, extracellular, matrix, proteoglycan, hyaluronic, hyaluronan, TGF, integrin, and TGFbeta.

### 4.5 Aging matreotype definition

To define the human aging matreotype, we performed a literature search and extracted the age association of all genes involved in forming the human matrisome. The inclusion criteria for the meta‐analysis require whole‐genome coverage for each study, availability of the full dataset including negative data, and that the publication has undergone peer‐review. If the datasets have not yet been subjected to a significance cutoff, we applied multiple testing corrected (Benjamini–Hochberg) threshold of 0.05 to each dataset if applicable. Tissue‐specific differences were not further investigated due to confounding in the meta‐analysis. Studies analyzing individual tissues were treated as separate sources. To define the aging matrisome, we acquired data from at least three sources implicating the gene in the aging process. Studies that offer directionality were further utilized to determine matrisome genes that were upregulated or downregulated with age using the same thresholds.

### 4.6 Collagen promoter‐driven GFP scoring

The strain LSD2002 P*col*‐*144*::*GFP* was used to assess age‐related decline in collagen‐144 expression. Animals were age‐synchronized by bleaching and made infertile by culturing at 25℃. NGM culturing plates were prepared with tretinoin (Sigma PHR1187), hyaluronic acid (Sigma H5388), chondroitin sulfate (Sigma 27042), metformin (Sigma D150959), glucosamine (Sigma G4875), and genistein (Changzhou Longterm Biotechnology Co., Ltd). Distribution scoring is based on intensity observed visual inspection, categorizing to none or very low, low, medium, or high intensity. For some experiments, animals were mounted onto 2% agar pads and pictures were taken with an upright fluorescent microscope (Tritech Research, model: BX‐51‐F). To separate the GFP signal from the autofluorescence of the gut, we used the microscope, settings, and triple‐band filterset as described by Teuscher (Teuscher & Ewald, 2018). The total intensity per animal, intensity [a.u.], is calculated from fluorescence images using FIJI. The detailed instruction and workflow are described in Supplementary File 1 and the python code in Supplementary File 2.

### 4.7 Lifespan measurements

Manual scoring of lifespan as previously described by Ewald et al. (2016). In brief, about 100 day‐2 adult *C. elegans* were picked to NGM plates containing the solvent either water or 0.1% dimethyl sulfoxide (DMSO) alone as control or tretinoin (Sigma PHR1187), hyaluronic acid (Sigma H5388), and chondroitin sulfate (Sigma 27042). Vitamin B12 (Sigma, PHR1234), chrysin (Santa Cruz Biotech, sc‐204686), and dapsone (Santa Cruz Biotech, sc‐203023A) lifespans were performed in 1.25% DMSO and 50 µM FUdR in 24‐well plates, 4 wells per condition and 30 worms per well, transferred at L4 developmental stage. Animals were classified as dead if they failed to respond to prodding. Exploded, bagged, burrowed, or animals that left the agar were excluded from the statistics. The estimates of survival functions were calculated using the product‐limit (Kaplan–Meier) method. The log‐rank (Mantel‐Cox) method was used to test the null hypothesis and calculate *P* values (JMP software v.14.1.0).

Additional Information can be found in the Extended Experimental Procedure in the Supporting Information available online.

## CONFLICT OF INTEREST

The authors have no competing interests to declare. Correspondence should be addressed to C. Y. E. The authors declare no conflict of interest. FW and FZ are employees of Mibelle Biochemistry.

## AUTHOR CONTRIBUTIONS

All authors participated in analyzing and interpreting the data. CYE and CS designed the experiments. CS established and performed *in* *‐* *silico* analysis. AD and PM translated compound names for list comparison and automated analysis. EJ, CS, AD, and CYE performed lifespan assays. EJ and CYE performed oxidative stress assays. EJ, AD, and SXL performed collagen expression assays. FW and FZ prepared and encapsulated compounds. CYE wrote the manuscript in consultation with the other authors.

## Supporting information

Figure S1

Figure S2

Figure S3

Figure S4

Figure S5

Figure S6

Figure S7

Figure S8

Figure S9

Figure S10

Figure S11

Figure S12

SupFile S1

SupFile S2

Supplementary Material

TableS1‐S8

## Data Availability

The data that support the findings of this study are available in GTEx at https://gtexportal.org/home/datasets and CMap ftp://ftp.broadinstitute.org/distribution/cmap and adopted from reference https://doi.org/10.1016/j.celrep.2019.03.044. Further data were derived from the following resources available in the public domain: http://matrisomeproject.mit.edu, https://genomics.senescence.info/genes/, http://geroprotectors.org. All sources and processed/re‐analyzed data are provided in Supplementary Tables in this study.

## References

[acel13441-bib-0001] Aliper, A., Belikov, A. V., Garazha, A., Jellen, L., Artemov, A., Suntsova, M., Ivanova, A., Venkova, L., Borisov, N., Buzdin, A., Mamoshina, P., Putin, E., Swick, A. G., Moskalev, A., & Zhavoronkov, A. (2016). In search for geroprotectors: In silico screening and in vitro validation of signalome‐level mimetics of young healthy state. Aging, 8, 2127–2152. 10.18632/aging.101047 27677171 PMC5076455

[acel13441-bib-0002] Bannister, C. A., Holden, S. E., Jenkins‐Jones, S., Morgan, C. L., Halcox, J. P., Schernthaner, G., Mukherjee, J., & Currie, C. J. (2014). Can people with type 2 diabetes live longer than those without? A comparison of mortality in people initiated with metformin or sulphonylurea monotherapy and matched, non‐diabetic controls. Diabetes, Obesity & Metabolism, 16, 1165–1173. 10.1111/dom.12354 25041462

[acel13441-bib-0003] Barardo, D., Thornton, D., Thoppil, H., Walsh, M., Sharifi, S., Ferreira, S., Anžič, A., Fernandes, M., Monteiro, P., Grum, T., Cordeiro, R., De‐Souza, E. A., Budovsky, A., Araujo, N., Gruber, J., Petrascheck, M., Fraifeld, V. E., Zhavoronkov, A., Moskalev, A., & de Magalhães, J. P. (2017). The DrugAge database of aging‐related drugs. Aging Cell, 16, 594–597. 10.1111/acel.12585 28299908 PMC5418190

[acel13441-bib-0004] Bell, G. A., Kantor, E. D., Lampe, J. W., Shen, D. D., & White, E. (2012). Use of glucosamine and chondroitin in relation to mortality. European Journal of Epidemiology, 27, 593–603. 10.1007/s10654-012-9714-6 22828954 PMC3557824

[acel13441-bib-0005] Calvert, S., Tacutu, R., Sharifi, S., Teixeira, R., Ghosh, P., & de Magalhães, J. P. (2016). A network pharmacology approach reveals new candidate caloric restriction mimetics in *C. elegans* . Aging Cell, 15, 256–266. 26676933 10.1111/acel.12432 PMC4783339

[acel13441-bib-0006] Chaturvedi, V., Kumar, J. U., Paithankar, K. R., Vanathi, P., & Sreedhar, A. S. (2011). Pharmacological inhibition of Hsp90 as a novel antitumor strategy to target cytoarchitecture through extracellular matrix signaling. Medicinal Chemistry, 7, 454–465. 21801149 10.2174/157340611796799212

[acel13441-bib-0007] Chen, G., Chen, H., Wang, C., Peng, Y., Sun, L., Liu, H., & Liu, F. (2012). Rapamycin ameliorates kidney fibrosis by inhibiting the activation of mTOR signaling in interstitial macrophages and myofibroblasts. PLoS One, 7, e33626. 10.1371/journal.pone.0033626 22470459 PMC3314672

[acel13441-bib-0008] Chung, C. L., Lawrence, I., Hoffman, M., Elgindi, D., Nadhan, K., Potnis, M., Jin, A., Sershon, C., Binnebose, R., Lorenzini, A., & Sell, C. (2019). Topical rapamycin reduces markers of senescence and aging in human skin: An exploratory, prospective, randomized trial. GeroScience, 41, 861–869. 10.1007/s11357-019-00113-y 31761958 PMC6925069

[acel13441-bib-0010] de Magalhães, J. P., Curado, J., & Church, G. M. (2009). Meta‐analysis of age‐related gene expression profiles identifies common signatures of aging. Bioinformatics, 25(7), 875–881. 10.1093/bioinformatics/btp073 19189975 PMC2732303

[acel13441-bib-0011] Dönertaş, H. M., Fuentealba, M., Partridge, L., & Thornton J. M. (2019). Identifying Potential Ageing‐Modulating Drugs In Silico. Trends in Endocrinology & Metabolism, 30(2), 118–131. 10.1016/j.tem.2018.11.005 30581056 PMC6362144

[acel13441-bib-0012] Dönertaş, H. M., Valenzuela, M. F., Partridge, L., & Thornton, J. M. (2018). Gene expression‐based drug repurposing to target aging. Aging Cell, 17, e12819. 10.1111/acel.12819 29959820 PMC6156541

[acel13441-bib-0014] Ewald, C. Y., Landis, J. N., Abate, J. P., Murphy, C. T., & Blackwell, T. K. (2015). Dauer‐independent insulin/IGF‐1‐signalling implicates collagen remodelling in longevity. Nature, 519, 97–101. 10.1038/nature14021 25517099 PMC4352135

[acel13441-bib-0015] Ewald, C. Y., & Marfil, V., Li Chris (2016). Alzheimer‐related protein APL ‐1 modulates lifespan through heterochronic gene regulation in Caenorhabditis elegans. Aging Cell, 15(8), 1051–1062. 10.1111/acel.12509 27557896 PMC5114704

[acel13441-bib-0013] Ewald, C. Y. (2020). The Matrisome during Aging and Longevity: A Systems‐Level Approach toward Defining Matreotypes Promoting Healthy Aging. Gerontology, 66(3), 266–274. 10.1159/000504295 31838471 PMC7214094

[acel13441-bib-0016] Fan, Y., Choi, T.‐H., Chung, J.‐H., Jeon, Y.‐K., & Kim, S. (2019). Hyaluronic acid‐cross‐linked filler stimulates collagen type 1 and elastic fiber synthesis in skin through the TGF‐β/Smad signaling pathway in a nude mouse model. Journal of Plastic, Reconstructive & Aesthetic Surgery: JPRAS, 72, 1355–1362. 10.1016/j.bjps.2019.03.032 31036501

[acel13441-bib-0017] Fuentealba, M., Dönertaş, H. M., Williams, R., Labbadia, J., Thornton, J. M., & Partridge, L. (2019). Using the drug‐protein interactome to identify anti‐ageing compounds for humans. L. M. Iakoucheva (Ed.). PLOS Computational Biology, 15(1), e1006639. 30625143 10.1371/journal.pcbi.1006639 PMC6342327

[acel13441-bib-0018] Griffiths, C., Russman, A. N., Majmudar, G., Singer, R. S., Hamilton, T. A., & Voorhees, J. J. (1993). Restoration of collagen formation in photodamaged human skin by tretinoin (retinoic acid). New England Journal of Medicine, 329, 530–535. 10.1056/NEJM199308193290803 8336752

[acel13441-bib-0019] Gueniche, A., & Castiel‐Higounenc, I. (2017). Efficacy of glucosamine sulphate in skin ageing: results from an ex vivo anti‐ageing model and a clinical trial. Skin Pharmacology and Physiology, 30, 36–41. 10.1159/000450832 28214837

[acel13441-bib-0020] Haes, W. D., Frooninckx, L., Assche, R. V., Smolders, A., Depuydt, G., Billen, J., Braeckman, B. P., Schoofs, L., & Temmerman, L. (2014). Metformin promotes lifespan through mitohormesis via the peroxiredoxin PRDX‐2. Proceedings of the National Academy of Sciences of the United States of America, 111, E2501–E2509. 24889636 10.1073/pnas.1321776111 PMC4066537

[acel13441-bib-0021] Hunter, M. C., O'Hagan, K. L., Kenyon, A., Dhanani, K. C. H., Prinsloo, E., & Edkins, A. L. (2014). Hsp90 binds directly to fibronectin (FN) and inhibition reduces the extracellular fibronectin matrix in breast cancer cells. PLoS One, 9, e86842. 10.1371/journal.pone.0086842 24466266 PMC3899338

[acel13441-bib-0022] Janssens, G. E., Lin, X.‐X., Millan‐Ariño, L., Kavšek, A., Sen, I., Seinstra, R. I., Stroustrup, N., Nollen, E. A. A., & Riedel, C. G. (2019). Transcriptomics‐Based Screening Identifies Pharmacological Inhibition of Hsp90 as a Means to Defer Aging. Cell Reports, 27(2), 467–480.e6. 10.1016/j.celrep.2019.03.044 30970250 PMC6459000

[acel13441-bib-0023] Komljenovic, A., Li, H., Sorrentino, V., Kutalik, Z., Auwerx, J., & Robinson‐Rechavi, M. (2019). Cross‐species functional modules link proteostasis to human normal aging R. Guigó, ed. PLoS Computational Biology, 15, e1007162. 10.1371/journal.pcbi.1007162 31269015 PMC6634426

[acel13441-bib-0024] Lamb, J., Crawford, E. D., Peck, D., Modell, J. W., Blat, I. C., Wrobel, M. J., Lerner, J., Brunet, J.‐P., Subramanian, A., Ross, K. N., Reich, M., Hieronymus, H., Wei, G., Armstrong, S. A., Haggarty, S. J., Clemons, P. A., Wei, R., Carr, S. A., Lander, E. S., & Golub, T. R. (2006). The Connectivity Map: Using gene‐expression signatures to connect small molecules, genes, and disease. Science, 313, 1929–1935. 10.1126/science.1132939 17008526

[acel13441-bib-0025] Lee, E. B., Ahn, D., Kim, B. J., Lee, S. Y., Seo, H. W., Cha, Y.‐S., Jeon, H., Eun, J. S., Cha, D. S., & Kim, D. K. (2015). Genistein from *Vigna angularis* extends lifespan in *Caenorhabditis elegans* . Biomolecules & Therapeutics, 23, 77–83. 10.4062/biomolther.2014.075 25593647 PMC4286753

[acel13441-bib-0026] Lephart, E. D., & Andrus, M. B. (2017). Human skin gene expression: Natural (trans) resveratrol versus five resveratrol analogs for dermal applications. Experimental Biology and Medicine (Maywood, N.J.), 242, 1482–1489. 10.1177/1535370217723628 PMC5648289 28750552

[acel13441-bib-0027] Li, P., Liang, M.‐L., Zhu, Y., Gong, Y.‐Y., Wang, Y., Heng, D., & Lin, L. (2014). Resveratrol inhibits collagen I synthesis by suppressing IGF‐1R activation in intestinal fibroblasts. World Journal of Gastroenterology, 20, 4648. 10.3748/wjg.v20.i16.4648 24782617 PMC4000501

[acel13441-bib-0028] Li, Z.‐H., Gao, X., Chung, V. C., Zhong, W.‐F., Fu, Q., Lv, Y.‐B., Wang, Z.‐H., Shen, D., Zhang, X.‐R., Zhang, P.‐D., Li, F.‐R., Huang, Q.‐M., Chen, Q., Song, W.‐Q., Wu, X.‐B., Shi, X.‐M., Kraus, V. B., Yang, X., & Mao, C. (2020). Associations of regular glucosamine use with all‐cause and cause‐specific mortality: A large prospective cohort study. Annals of the Rheumatic Diseases, 79, annrheumdis‐2020‐217176. 10.1136/annrheumdis-2020-217176 PMC7286049 32253185

[acel13441-bib-0029] Lippiello, L. (2007). Collagen synthesis in tenocytes, ligament cells and chondrocytes exposed to a combination of glucosamine HCl and chondroitin sulfate. Evidence‐Based Complementary and Alternative Medicine, 4, 219–224. 10.1093/ecam/nel081 17549239 PMC1876619

[acel13441-bib-0030] Liu, H., Guo, M., Xue, T., Guan, J., Luo, L., & Zhuang, Z. (2016). Screening lifespan‐extending drugs in Caenorhabditis elegans via label propagation on drug‐protein networks. BMC Systems Biology, 10(54), 10.1186/s12918-016-0362-4 PMC5260106 28155715

[acel13441-bib-0009] Lonsdale, J., Thomas, J., Salvatore, M., Phillips, R., Lo, E., Shad, S., Hasz, R., Walters, G., Garcia, F., Young, N., Foster, B., Moser, M., Karasik, E., Gillard, B., Ramsey, K., Sullivan, S., Bridge, J., Magazine, H., Syron, J., … Moore, H. F. (2013). The Genotype‐Tissue Expression (GTEx) project. Nature Genetics, 45(6), 580–585. 10.1038/ng.2653 23715323 PMC4010069

[acel13441-bib-0031] Lu, Y., Brommer, B., Tian, X., Krishnan, A., Meer, M., Wang, C., Vera, D. L., Zeng, Q., Yu, D., Bonkowski, M. S., Yang, J.‐H., Zhou, S., Hoffmann, E. M., Karg, M. M., Schultz, M. B., Kane, A. E., Davidsohn, N., Korobkina, E., Chwalek, K., … Sinclair, D. A. (2020). Reprogramming to recover youthful epigenetic information and restore vision. Nature, 588(7836), 124–129. 10.1038/s41586-020-2975-4 33268865 PMC7752134

[acel13441-bib-0032] Matori, H., Umar, S., Nadadur, R. D., Sharma, S., Partow‐Navid, R., Afkhami, M., Amjedi, M., & Eghbali, M. (2012). Genistein, a soy phytoestrogen, reverses severe pulmonary *Hypertension* and prevents right heart failure in rats. Hypertension, 60, 425–430. 10.1161/HYPERTENSIONAHA.112.191445 22753213 PMC4252152

[acel13441-bib-0033] Moskalev, A., Chernyagina, E., de Magalhães, J. P., Barardo, D., Thoppil, H., Shaposhnikov, M., Budovsky, A., Fraifeld, V. E., Garazha, A., Tsvetkov, V., Bronovitsky, E., Bogomolov, V., Scerbacov, A., Kuryan, O., Gurinovich, R., Jellen, L. C., Kennedy, B., Mamoshina, P., Dobrovolskaya, E., … Zhavoronkov, A. (2015). Geroprotectors.org: a new, structured and curated database of current therapeutic interventions in aging and age‐related disease. Aging, 7(9), 616–628. 10.18632/aging.100799 26342919 PMC4600621

[acel13441-bib-0034] Mukherjee, S., Date, A., Patravale, V., Korting, H. C., Roeder, A., & Weindl, G. (2006). Retinoids in the treatment of skin aging: An overview of clinical efficacy and safety. Clinical Interventions in Aging, 1, 327–348. 10.2147/ciia.2006.1.4.327 18046911 PMC2699641

[acel13441-bib-0035] Naba, A., Clauser, K. R., Ding, H., Whittaker, C. A., Carr, S. A., & Hynes, R. O. (2016). The extracellular matrix: Tools and insights for the “omics” era. Matrix Biology, 49, 10–24. 10.1016/j.matbio.2015.06.003 26163349 PMC5013529

[acel13441-bib-0036] Partridge, L., Deelen, J., & Slagboom, P. E. (2018). Facing up to the global challenges of ageing. Nature, 561, 45–56. 10.1038/s41586-018-0457-8 30185958

[acel13441-bib-0037] Polito, F., Marini, H., Bitto, A., Irrera, N., Vaccaro, M., Adamo, E. B., Micali, A., Squadrito, F., Minutoli, L., & Altavilla, D. (2012). Genistein aglycone, a soy‐derived isoflavone, improves skin changes induced by ovariectomy in rats. British Journal of Pharmacology, 165(4), 994–1005. 10.1111/j.1476-5381.2011.01619.x 21827449 PMC3312494

[acel13441-bib-0038] Pryor, R., & Cabreiro, F. (2015). Repurposing metformin: an old drug with new tricks in its binding pockets. Biochemical Journal, 471(3), 307–322. 10.1042/bj20150497 PMC4613459 26475449

[acel13441-bib-0039] Riera, C. E., & Dillin, A. (2015). Can aging be 'drugged'?. Nature Medicine, 21(12), 1400–1405. 10.1038/nm.4005 26646496

[acel13441-bib-0040] Socovich, A. M., & Naba, A. (2019). The cancer matrisome: From comprehensive characterization to biomarker discovery. Seminars in Cell & Developmental Biology, 89, 157–166. 10.1016/j.semcdb.2018.06.005 29964200

[acel13441-bib-0041] Statzer, C., & Ewald, C. Y. (2020). The extracellular matrix phenome across species. Matrix Biology plus, 8, 100039. 10.1016/j.mbplus.2020.100039 33543035 PMC7852310

[acel13441-bib-0042] Stroustrup, N., Anthony, W. E., Nash, Z. M., Gowda, V., Gomez, A., López‐Moyado, I. F., Apfeld, J., & Fontana, W. (2016). The temporal scaling of *Caenorhabditis elegans* ageing. Nature, 530, 103–107. 10.1038/nature16550 26814965 PMC4828198

[acel13441-bib-0043] Taha, I. N., & Naba, A. (2019). Exploring the extracellular matrix in health and disease using proteomics. Essays in Biochemistry, 63(3), 417–432. 10.1042/ebc20190001 31462529

[acel13441-bib-0044] Tarkhov, A. E., Alla, R., Ayyadevara, S., Pyatnitskiy, M., Menshikov, L. I., Shmookler Reis, R. J., & Fedichev, P. O. (2019). A universal transcriptomic signature of age reveals the temporal scaling of Caenorhabditis elegans aging trajectories. Scientific Reports, 9(1), 10.1038/s41598-019-43075-z PMC6517414 31089188

[acel13441-bib-0045] Teuscher, A., & Ewald, C. (2018). Overcoming Autofluorescence to Assess GFP Expression During Normal Physiology and Aging in Caenorhabditis elegans. BIO‐PROTOCOL, 8(14), 1–17. 10.21769/bioprotoc.2940 PMC6067662 30073182

[acel13441-bib-0046] Teuscher, A. C., Jongsma, E., Davis, M. N., Statzer, C., Gebauer, J. M., Naba, A., & Ewald, C. Y. (2019). The in‐silico characterization of the Caenorhabditis elegans matrisome and proposal of a novel collagen classification. Matrix Biology Plus, 1, 100001. 10.1016/j.mbplus.2018.11.001 33543001 PMC7852208

[acel13441-bib-0047] Tyshkovskiy, A., Bozaykut, P., Borodinova, A. A., Gerashchenko, M. V., Ables, G. P., Garratt, M., Khaitovich, P., Clish, C. B., Miller, R. A., & Gladyshev, V. N. (2019). Identification and Application of Gene Expression Signatures Associated with Lifespan Extension. Cell Metabolism, 30(3), 573–593.e8. 10.1016/j.cmet.2019.06.018 31353263 PMC6907080

[acel13441-bib-0048] Wang, F., Garza, L. A., Kang, S., Varani, J., Orringer, J. S., Fisher, G. J., & Voorhees, J. J. (2007). In Vivo Stimulation of De Novo Collagen Production Caused by Cross‐linked Hyaluronic Acid Dermal Filler Injections in Photodamaged Human Skin. Archives of Dermatology, 143(2), 10.1001/archderm.143.2.155 17309996

[acel13441-bib-0049] Weimer, S., Priebs, J., Kuhlow, D., Groth, M., Priebe, S., Mansfeld, J., Merry, T. L., Dubuis, S., Laube, B., Pfeiffer, A. F., Schulz, T. J., Guthke, R., Platzer, M., Zamboni, N., Zarse, K., & Ristow, M. (2014). D‐Glucosamine supplementation extends life span of nematodes and of ageing mice. Nature Communications, 5, 3563. 10.1038/ncomms4563 PMC3988823 24714520

[acel13441-bib-0050] Zeng, L., Yang, J., Peng, S., Zhu, J., Zhang, B., Suh, Y., & Tu, Z. (2020). Transcriptome analysis reveals the difference between “healthy” and “common” aging and their connection with age‐related diseases. Aging Cell, 19, e13121. 10.1111/acel.13121 32077223 PMC7059150

