# Supplementary figures and images for: Youthful and age‐related matreotypes predict drugs promoting longevity
Source: Aging Cell. 2021 Aug 4;20(9):e13441. doi: 10.1111/acel.13441 (PMC8441316; doi:10.1111/acel.13441)

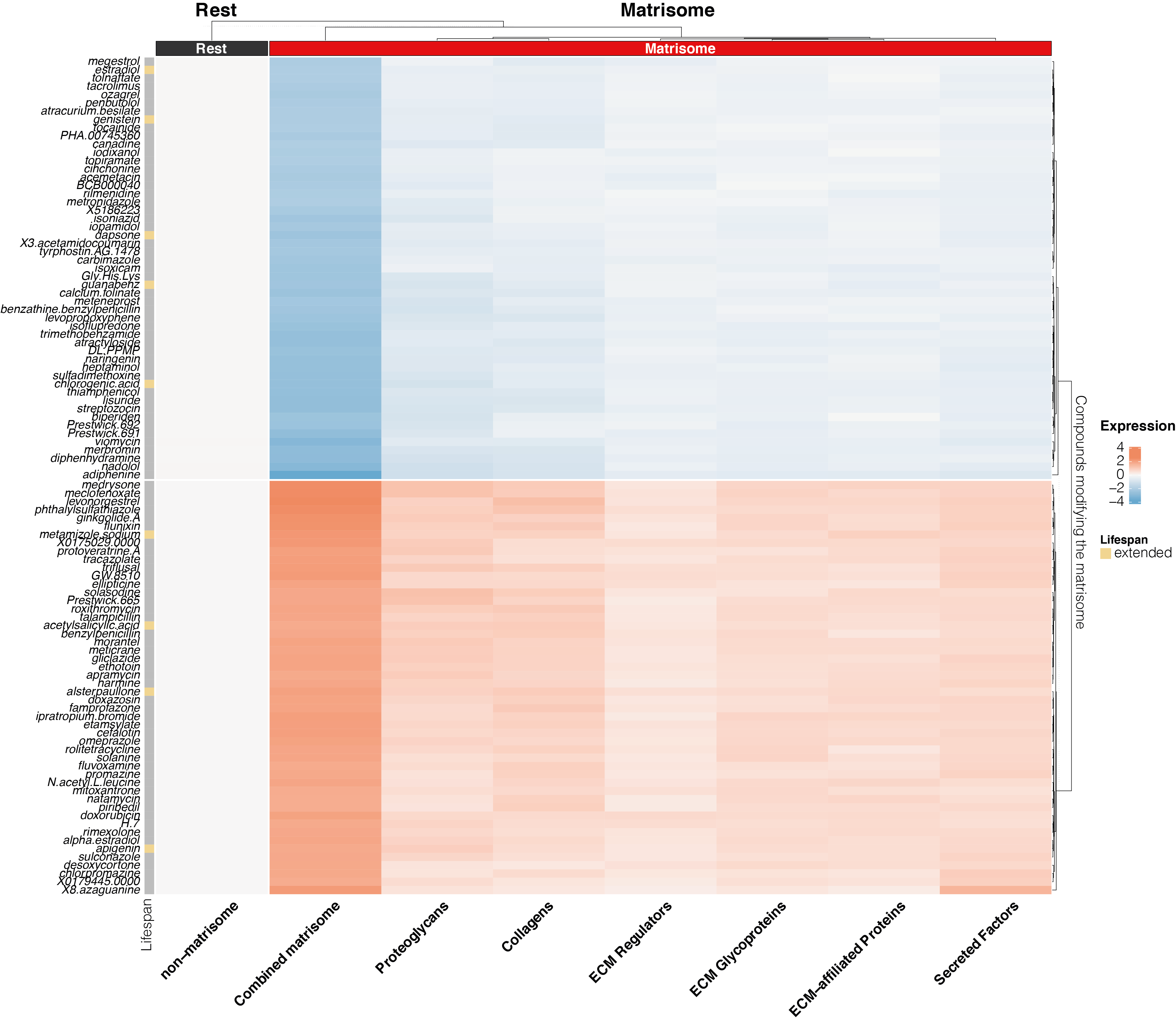

Supplement: Supplementary file 1 — Figure S1 [file ACEL-20-e13441-s001.png]

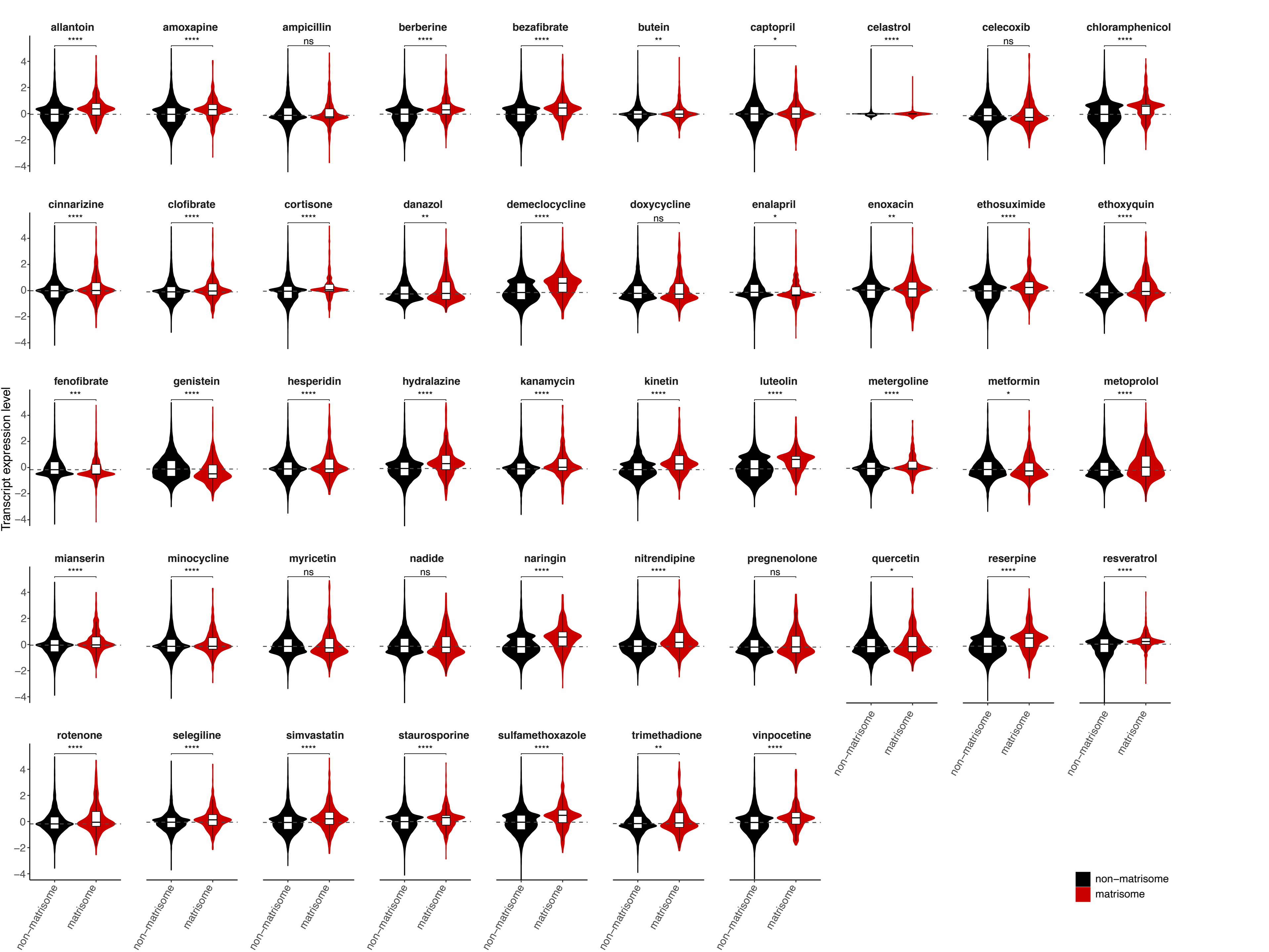

Supplement: Supplementary file 2 — Figure S2 [file ACEL-20-e13441-s012.pdf]

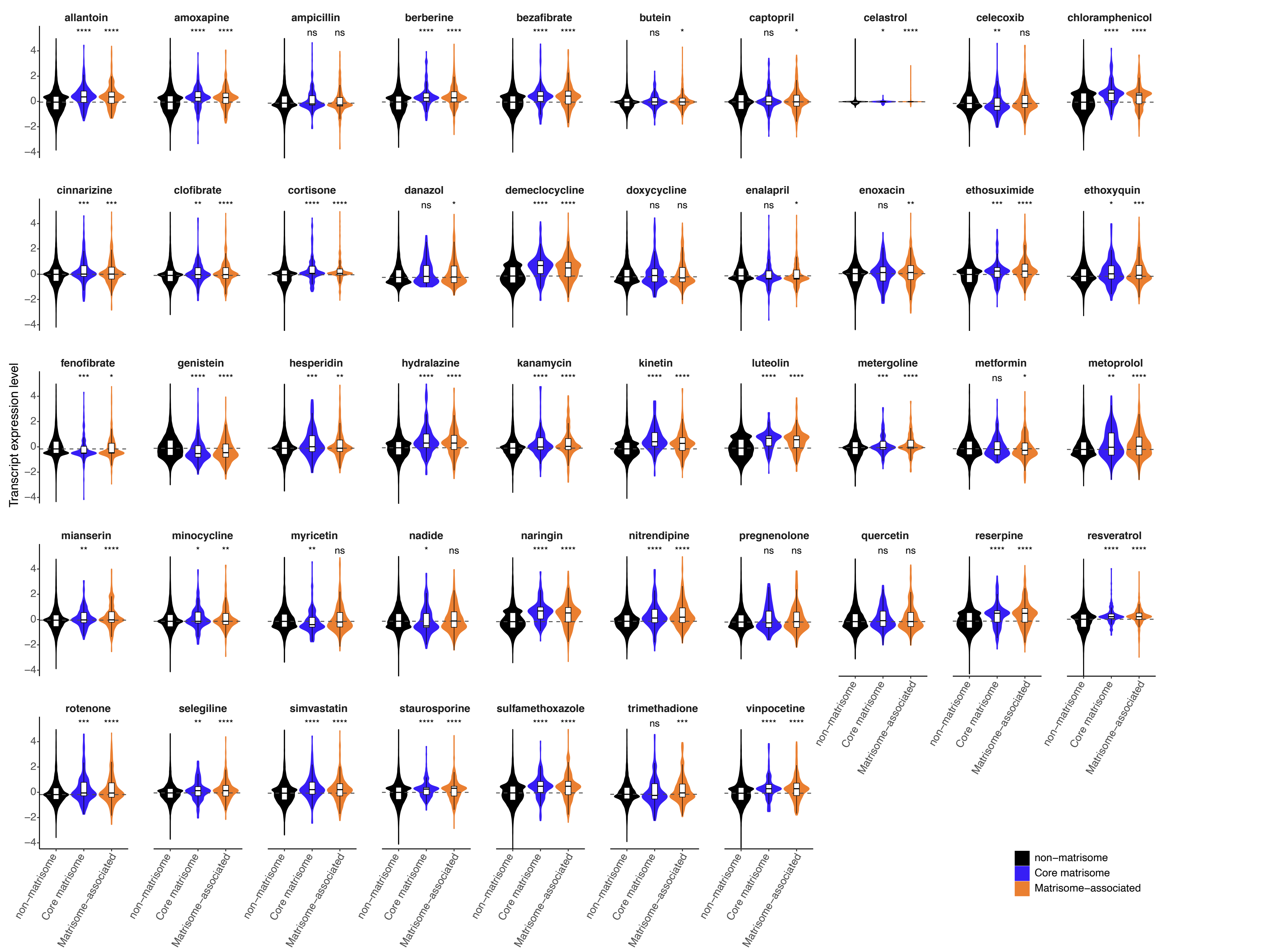

Supplement: Supplementary file 3 — Figure S3 [file ACEL-20-e13441-s002.pdf]

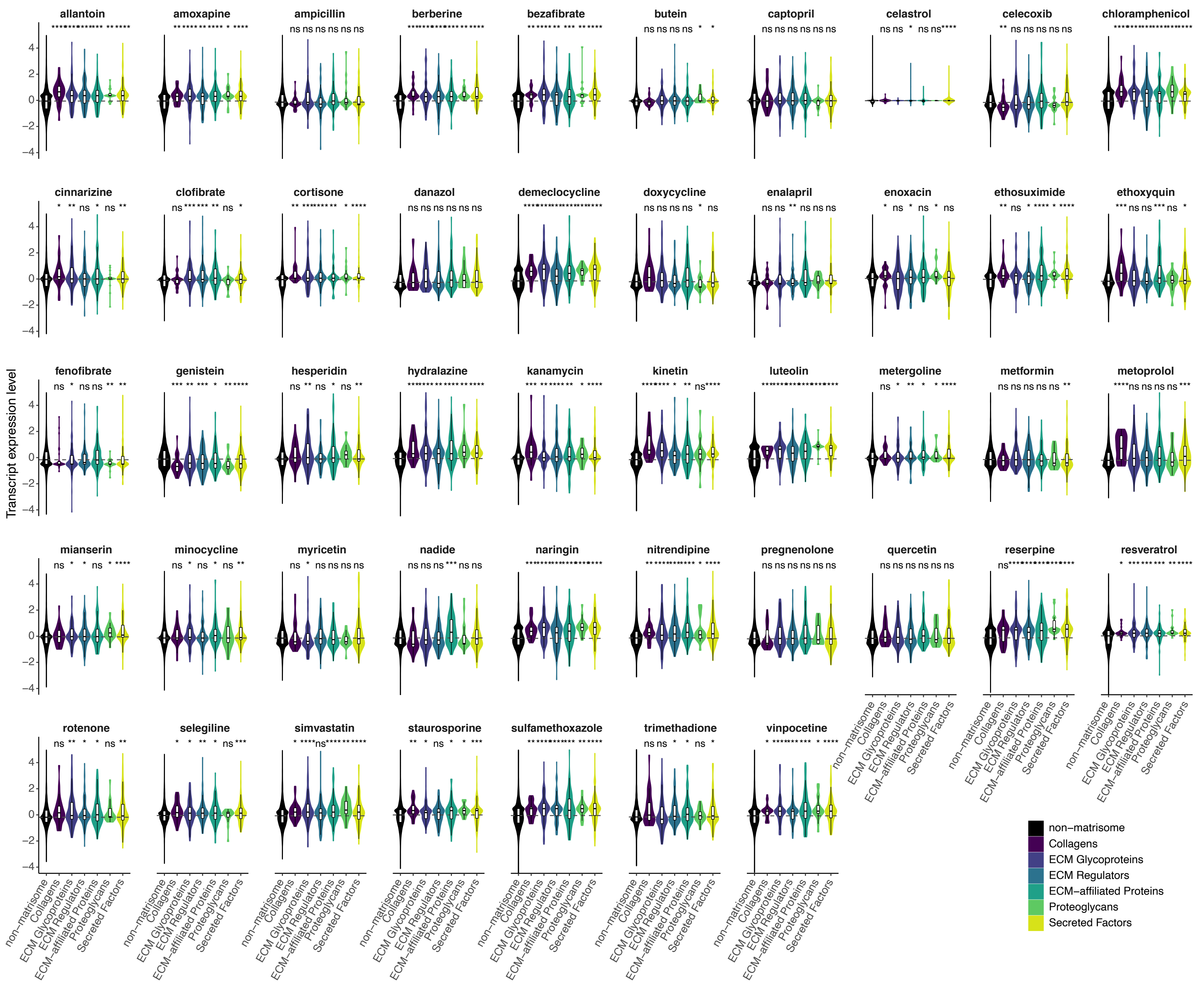

Supplement: Supplementary file 4 — Figure S4 [file ACEL-20-e13441-s010.pdf]

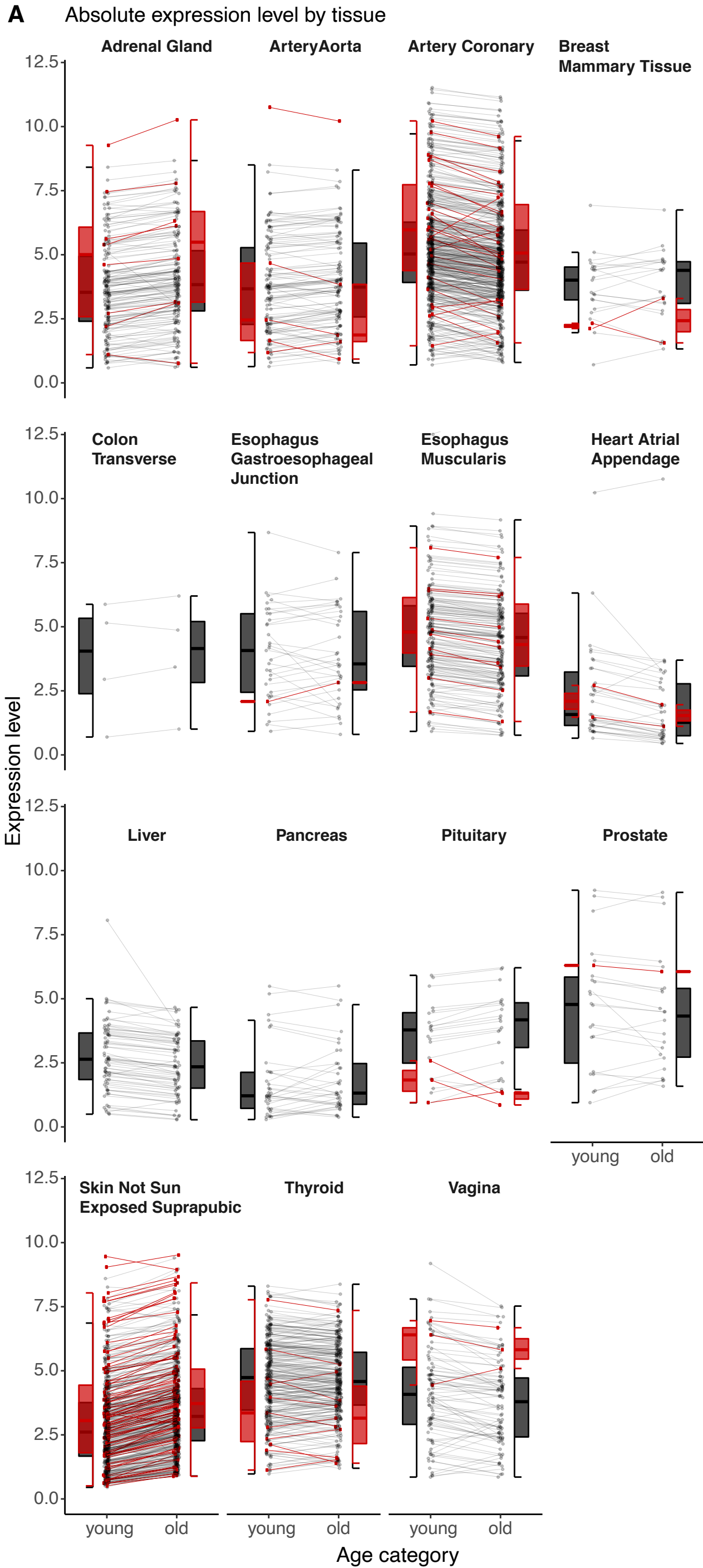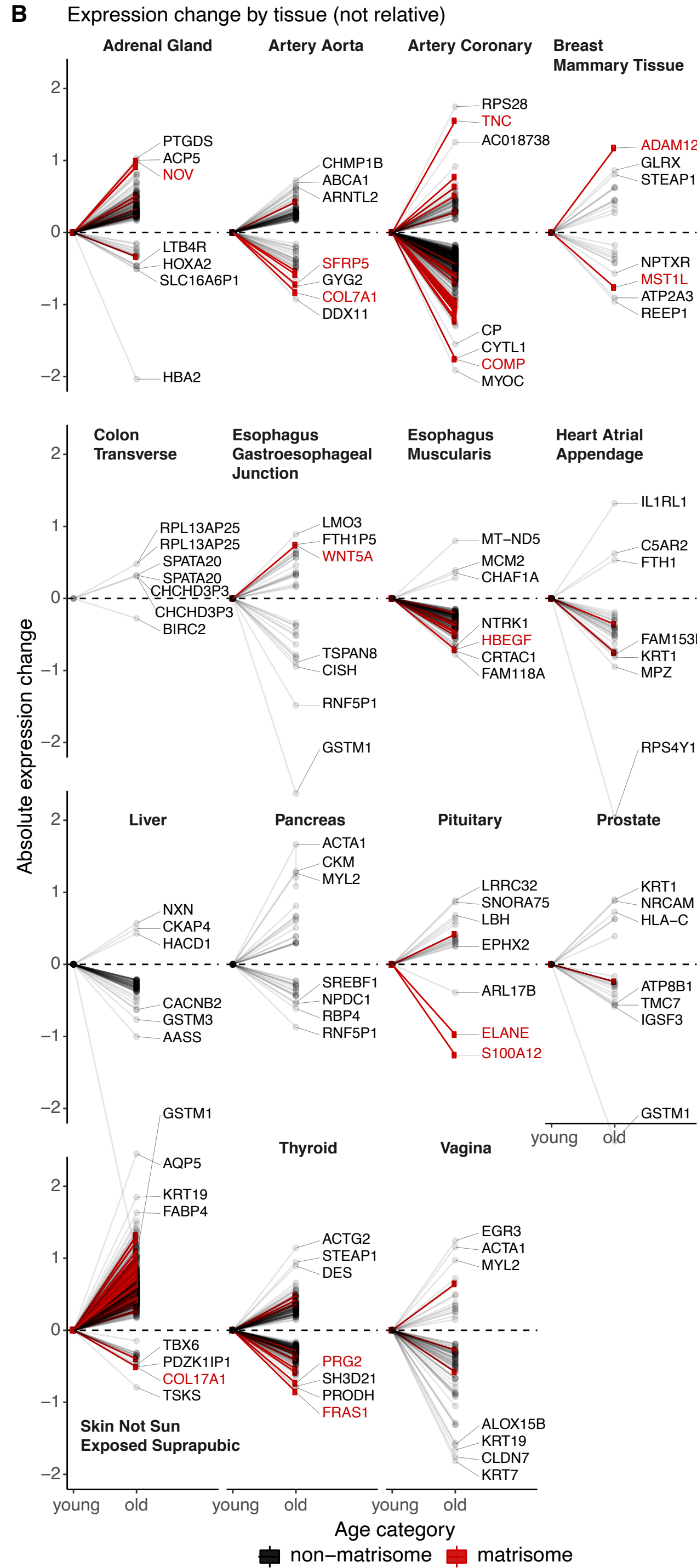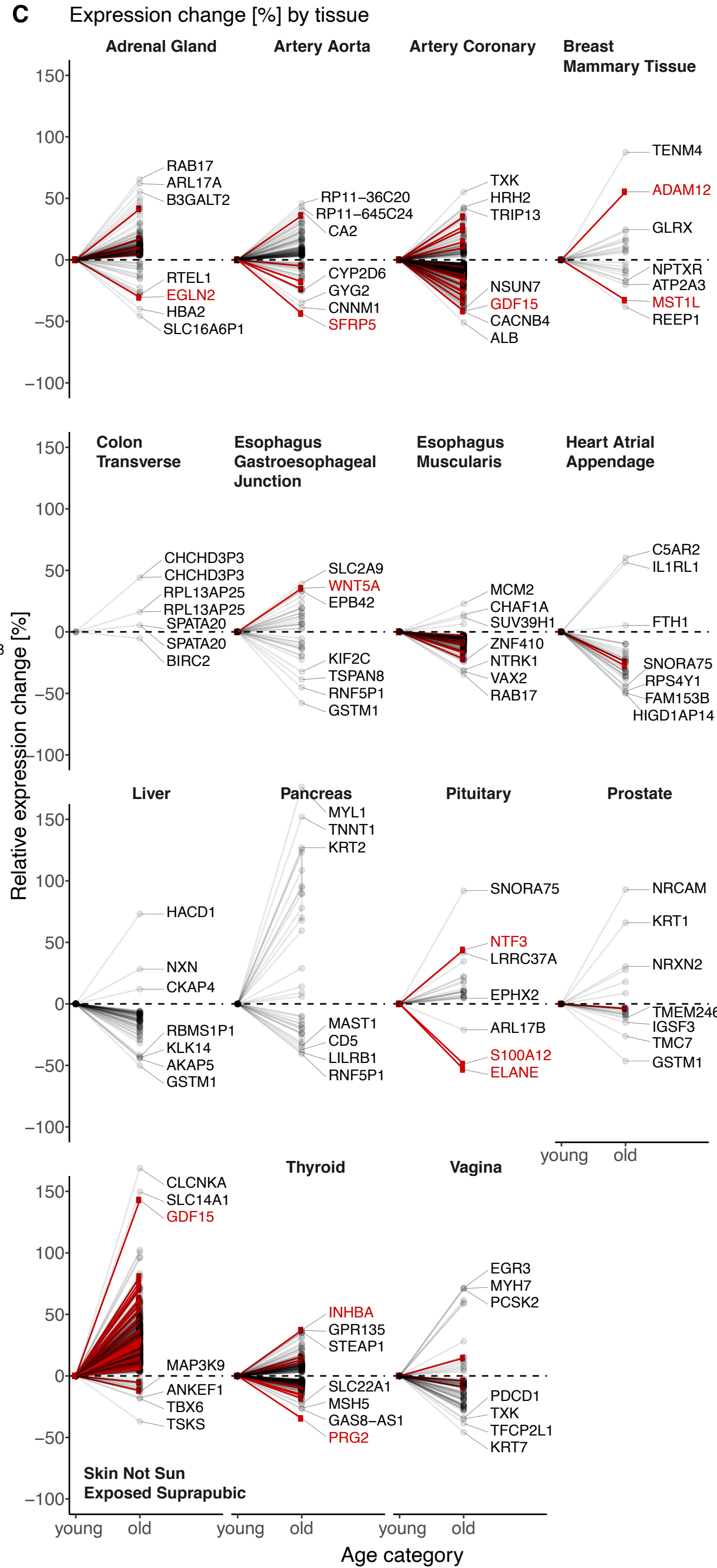

Supplement: Supplementary file 5 — Figure S5 [file ACEL-20-e13441-s006.pdf]

Expression change [%] by tissue and matrisome category

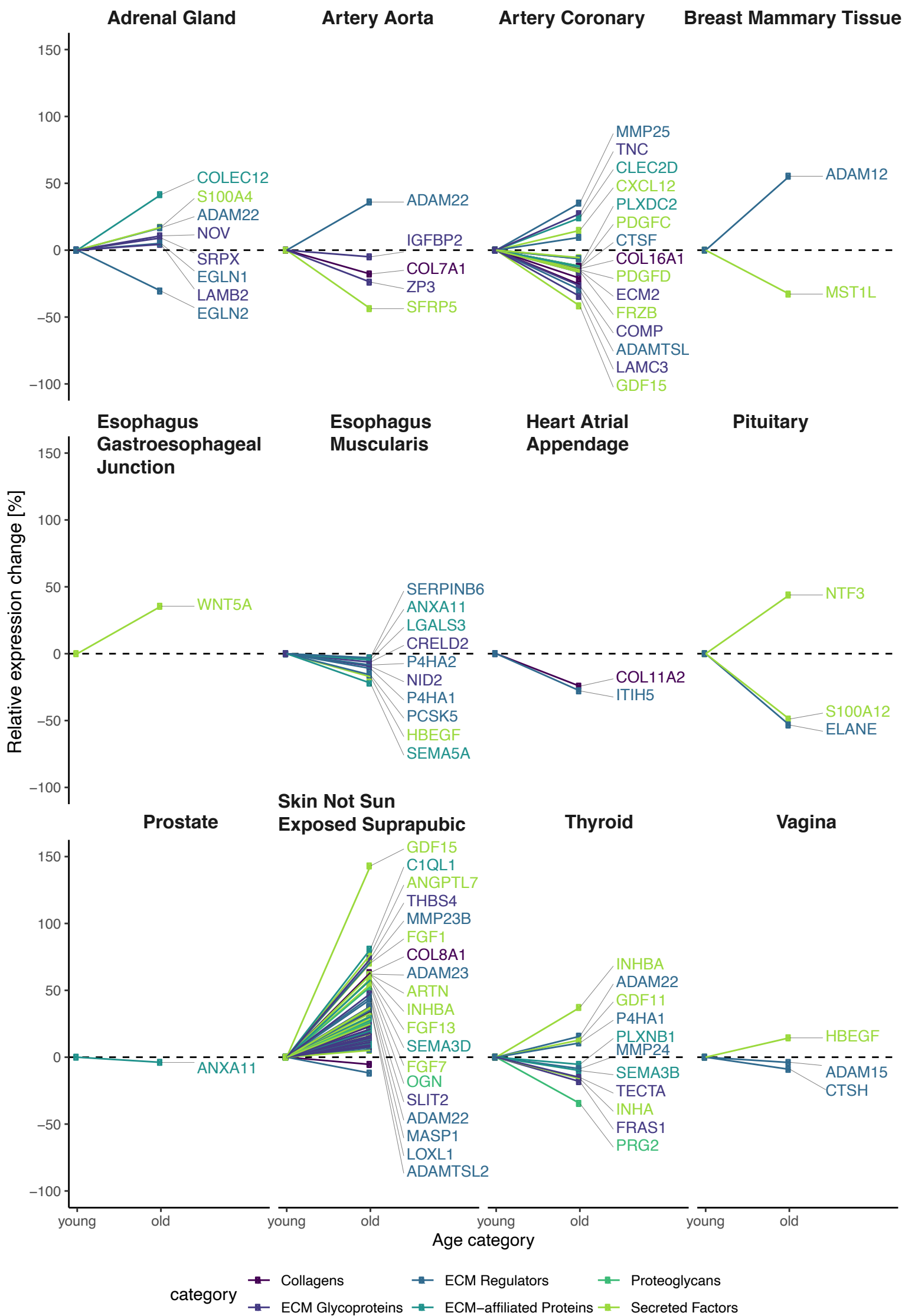

Supplement: Supplementary file 6 — Figure S6 [file ACEL-20-e13441-s003.pdf]

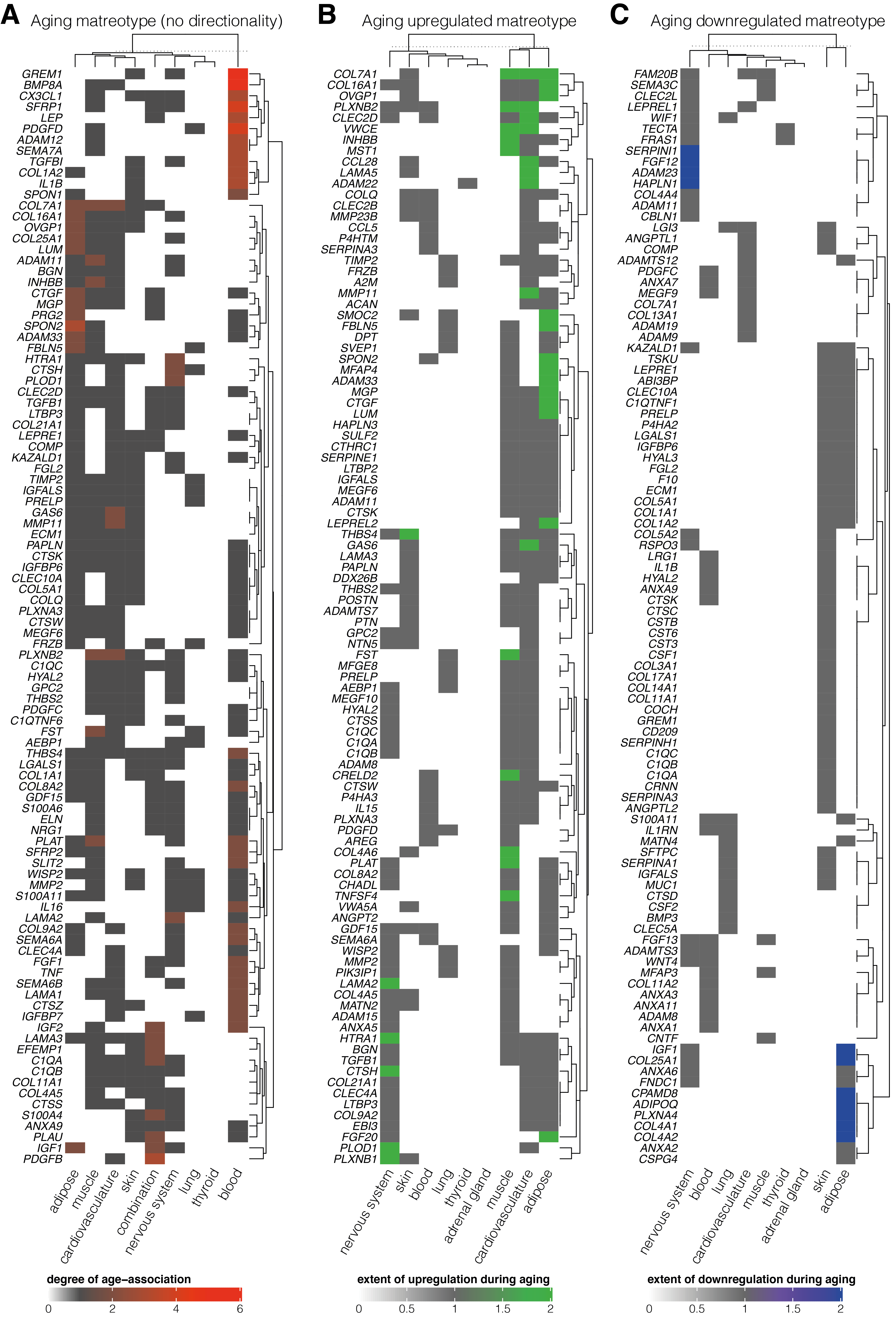

Supplement: Supplementary file 8 — Figure S8 [file ACEL-20-e13441-s013.png]

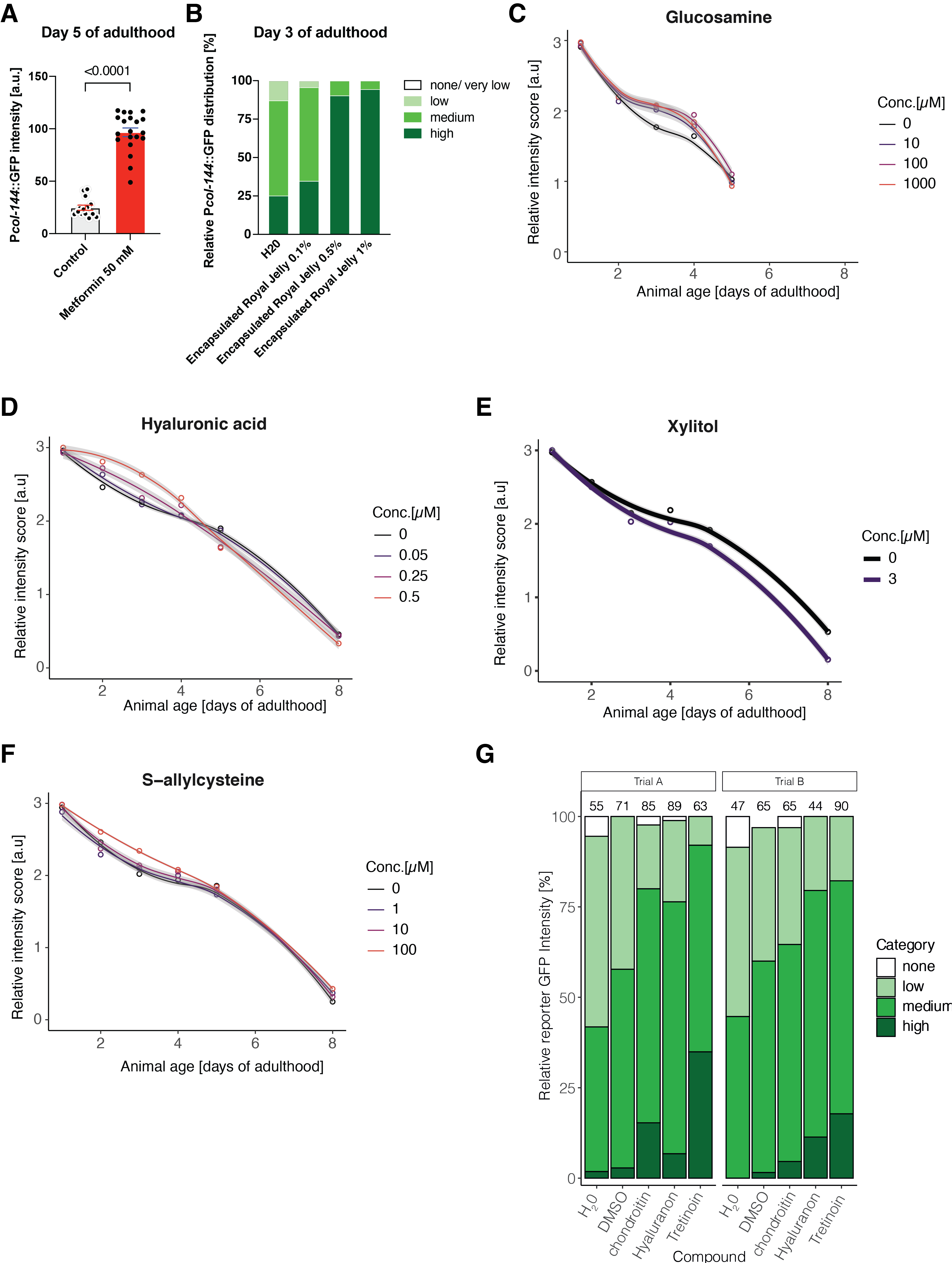

Supplement: Supplementary file 10 — Figure S10 [file ACEL-20-e13441-s004.png]

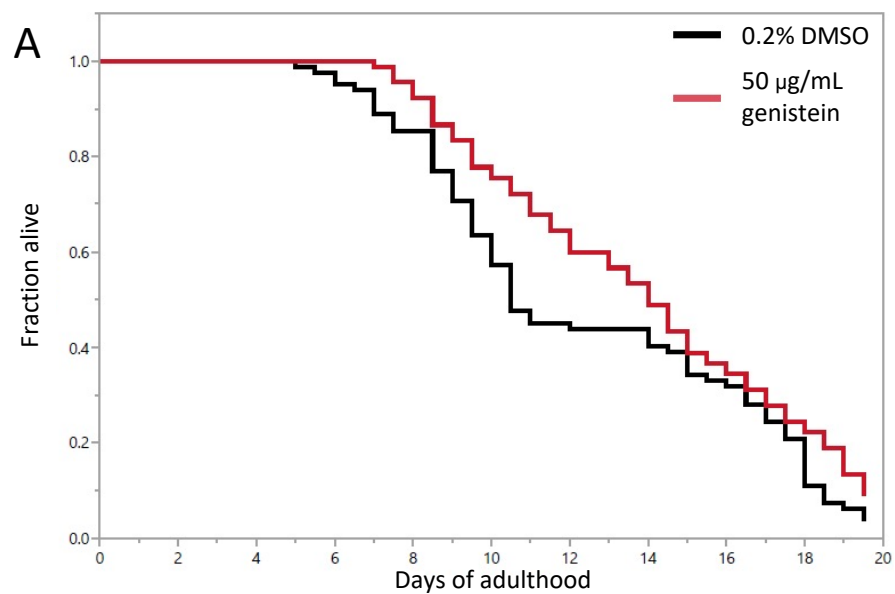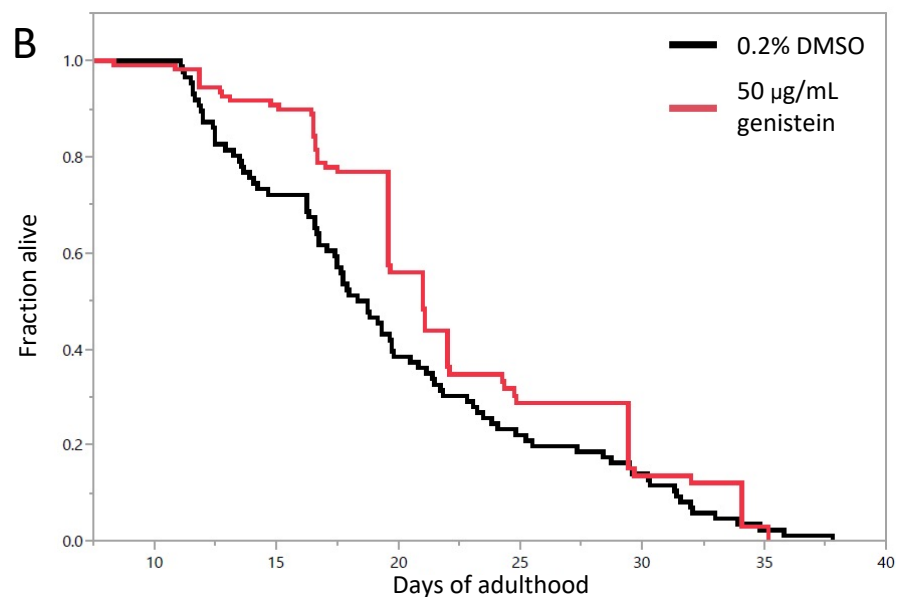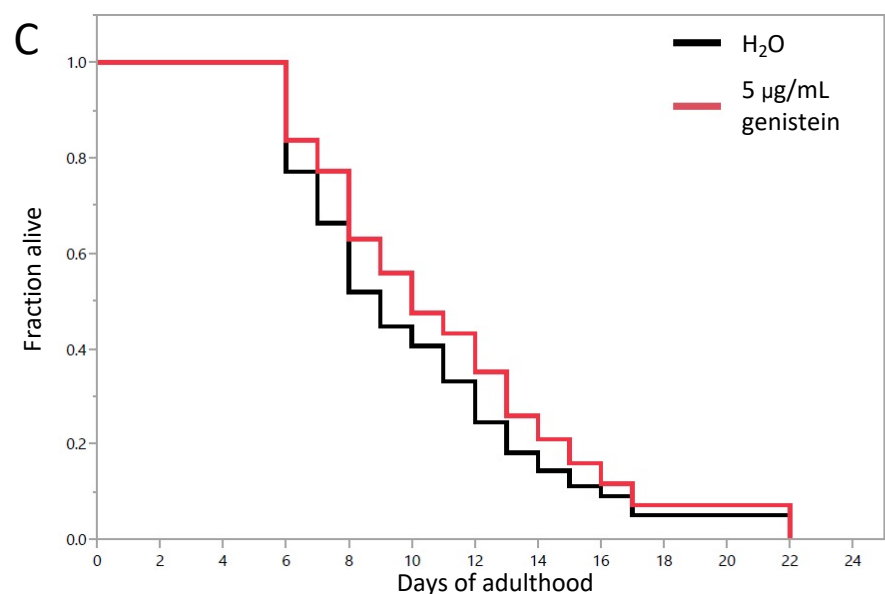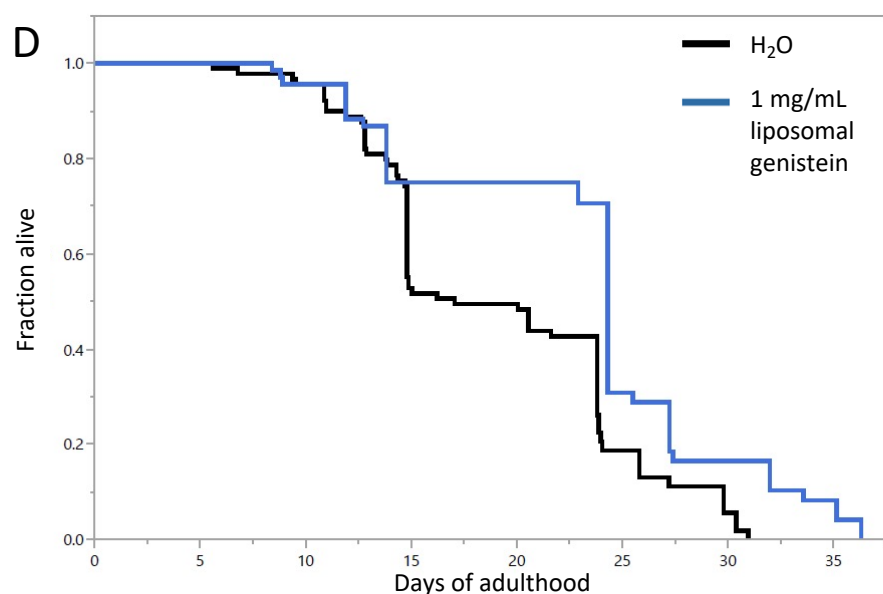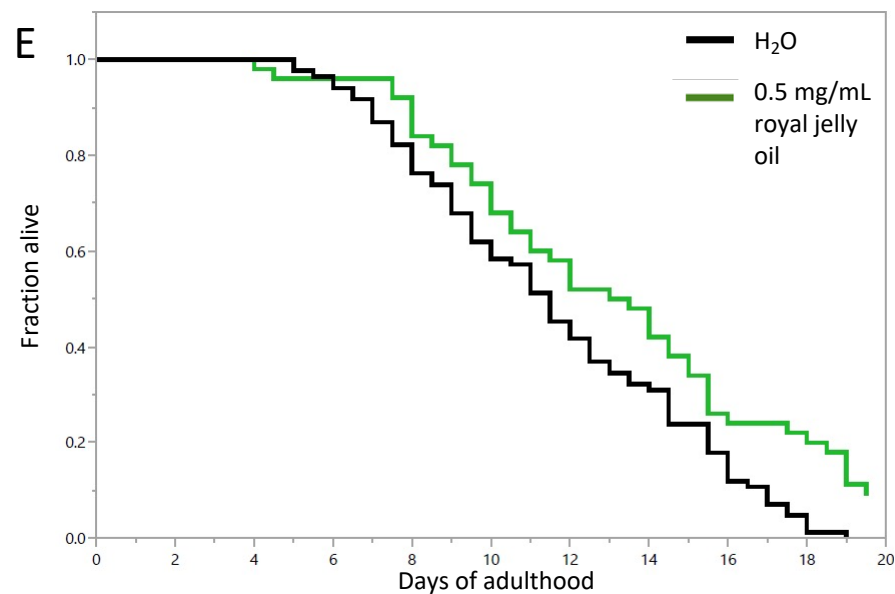

Supplement: Supplementary file 11 — Figure S11 [file ACEL-20-e13441-s005.pdf]

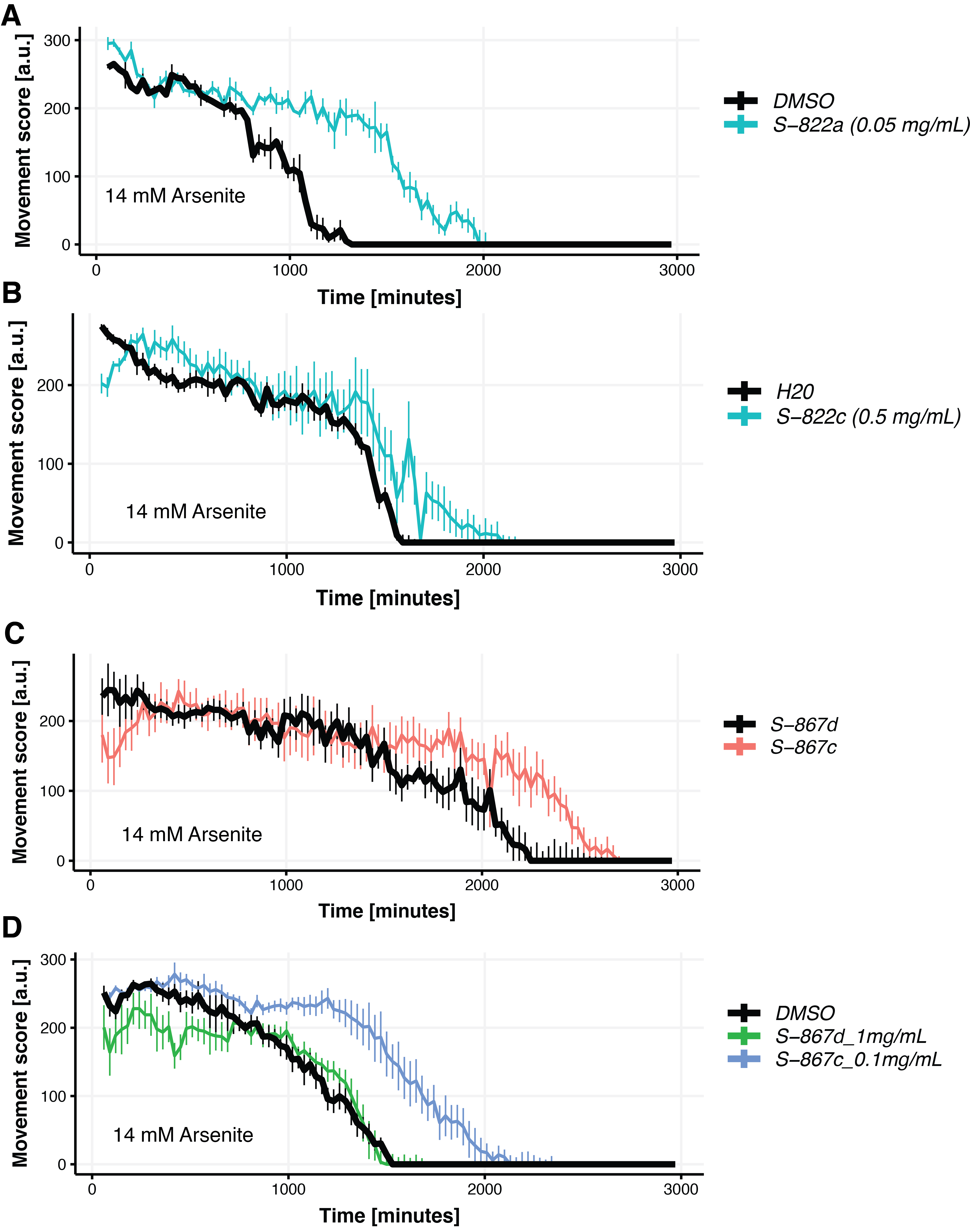

Supplement: Supplementary file 12 — Figure S12 [file ACEL-20-e13441-s011.png]
