# Supplementary material for: Youthful and age‐related matreotypes predict drugs promoting longevity
Source: Aging Cell. 2021 Aug 4;20(9):e13441. doi: 10.1111/acel.13441 (PMC8441316; doi:10.1111/acel.13441)
Supplement: Supplementary file 9 — Figure S9 [file ACEL-20-e13441-s014.pdf]

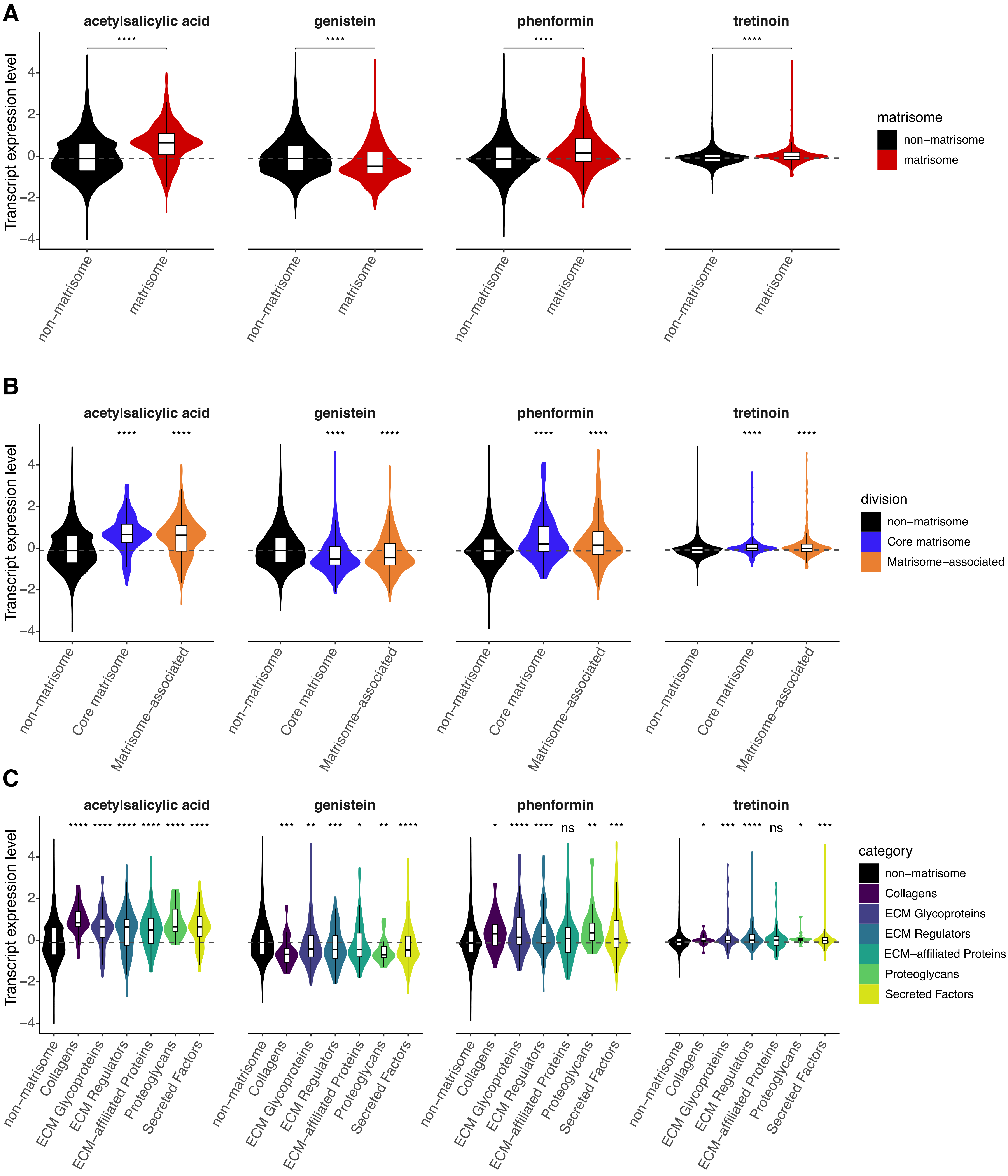
A
acetylsalicylic acid
genistein
phenformin
tretinoin
****
Transcript expression level
4
2
0
−2
−4
non−matrisome
matrisome
B
acetylsalicylic acid
genistein
phenformin
tretinoin
****
Transcript expression level
non−matrisome
Core matrisome
Matrisome−associated
division
C
acetylsalicylic acid
**** **** **** **** **** ****
genistein
*** ** *** * ** ****
phenformin
* **** **** ns ** ***
tretinoin
* *** **** ns * ***
Transcript expression level
category
non−matrisome
Collagens
ECM Glycoproteins
ECM Regulators
ECM−affiliated Proteins
Proteoglycans
Secreted Factors
