# Supplementary material for: Youthful and age‐related matreotypes predict drugs promoting longevity
Source: Aging Cell. 2021 Aug 4;20(9):e13441. doi: 10.1111/acel.13441 (PMC8441316; doi:10.1111/acel.13441)
Supplement: Supplementary file 13 — SupFile S1 [file ACEL-20-e13441-s009.pdf]

Workflow analysis for quantifying GFP intensity – autofluorescence omitted.

How to operate the program

1. Install ImageJ and place the program in the same folder as your ImageJ.exe.
2. Open ImageJ
3. Go to File -> open -> and select the program "GFPminusAutofluorescence.py"

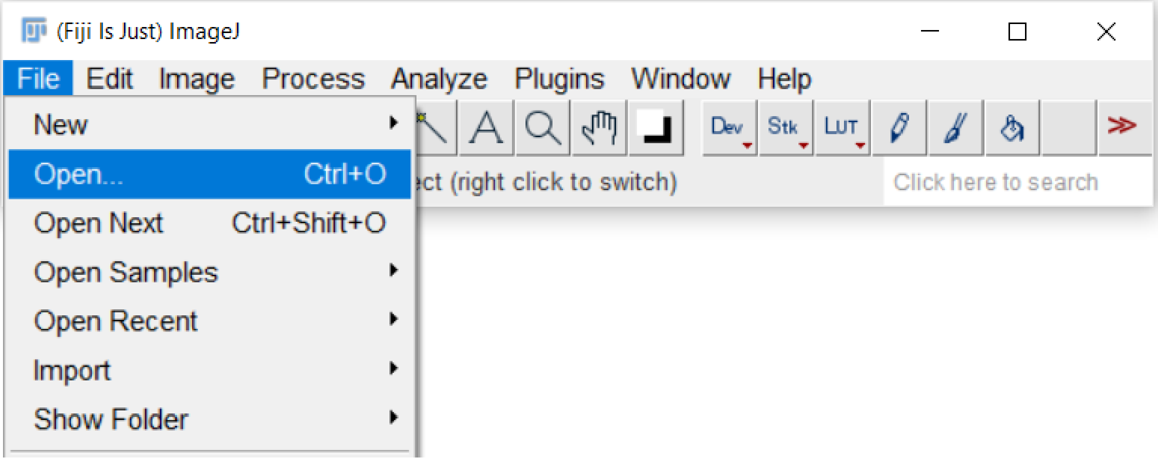

4. A new window opens, go to Language and select "Python".

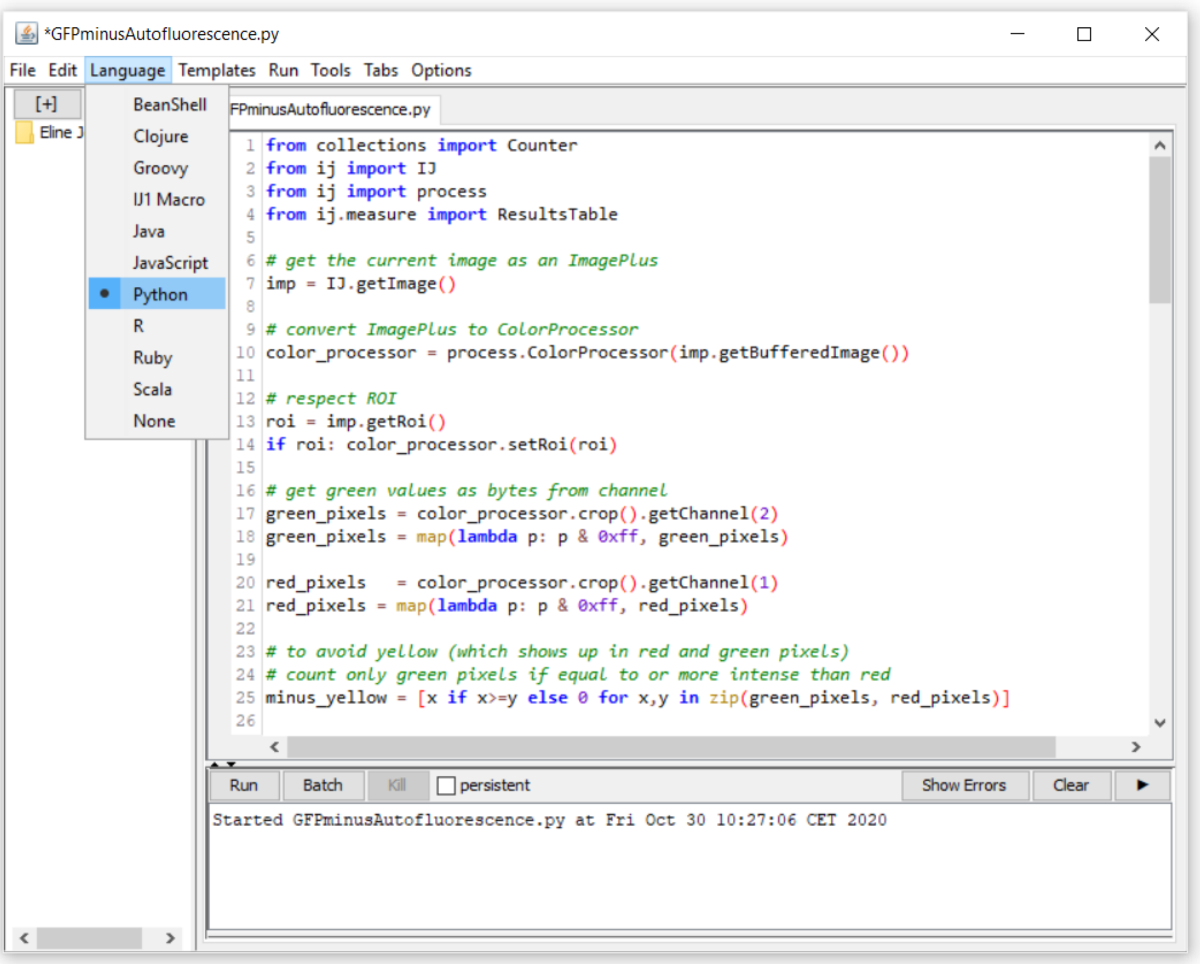

5. Open an image, select your area of interest, and press “run”.
6. The output appears in a new window, starting a table with your consecutive measurements.

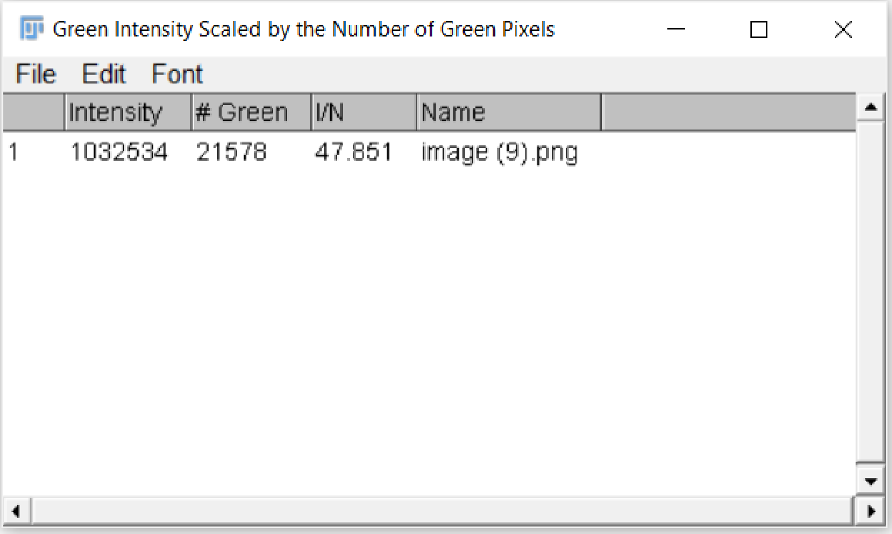

7. Under “Intensity” is the total intensity of green pixels found in your ROI. Under “# Green” is the number of green pixels in your ROI. Under “I/N” is the total intensity divided by the number of green pixels in your ROI. Under “Name” is the name of your file.
8. The content of the table can be exported by copy-paste to excel.

The code – explained

The code is designed to measure the GFP intensity in transgenic *C. elegans* animals and subtracting the autofluorescence of the gut. This program might be useful or easily adaptable to measure and subtract Red, Green, or Blue signals for other purposes.

Every line started with “#” is intended for human eyes and is not run by the program. It suits to explain the next piece of code, so the reader knows where to change the code to adapt it to one’s wishes.

First, the digital image is collected, and the area of interest is selected. The intensities for the green and red channels are collected. Furthermore, the range of intensities is corrected from range -128 to 127, to a range of 0 to 255.

To measure only the green signal was the main challenge of the code. Images are made of red, green, and blue signals; but the autofluorescence of the animal gut shows up in yellow. To remove this yellow, we initially split the .tiff image into the three channels (red, green, blue) and subtracted the red from the green channel. However, due to the nature of the green channel always containing red (especially with high intensities), we lost the green signal, especially when the signal was intense. Next, we noticed that as a green pixel gets brighter, not only is there an increase in green signal, there is also an increase in the blue signal; this is due to the algorithm the camera uses for de-mosaicking and how the camera converts it’s colors to a digital value. So, we tried to add the blue channel back in with the green after removing the red. However, this led to an overall enhancement of the green signal.

Finally, we wondered what the yellow signal consists of in the .tiff image. Pure yellow should, in theory, have equal amounts of red and green in the three color channels. However, the autofluorescence signal in our .tiff images always shows higher red than green or blue intensities. Hence, we concluded to only process the green intensities when it is more intense than the intensity of the red channel. Intensities, whereby the red is as strong or stronger than the green channel, are omitted from processing (Figure 1). If we would omit the signal where red=green intensity, we lose the intensity of saturated pixels; hence, these are not omitted.

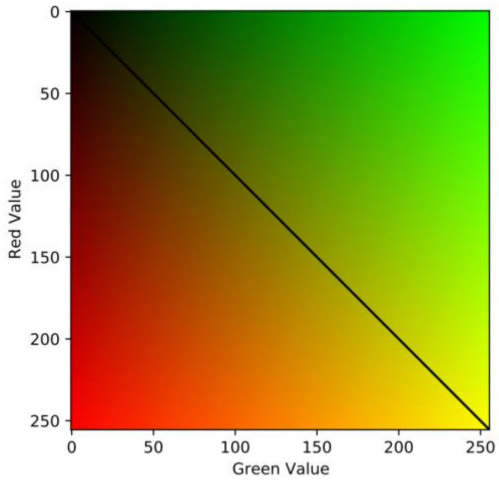

Figure 1. Cut-off for red > green. Any data where red > green is omitted. Only signals above the line are processed.

Under ### VISUAL TEST ###, a visual test for the removal of autofluorescence can be run to see if the current subtraction is according to one's needs. This requires only the removal of the "#" in front of lines 31-35, 36-38, 41-45.

A python collection counter can be used to count the number of times each pixel intensity is observed. Remove the "#" in front of line 63 to see the number of pixels per intensity. To get the total intensity, the intensity per pixel is multiplied by the number of times it was observed and added up for all pixels with intensities above 6. In this code, an intensity below 6 (five or lower) is assumed to be background noise and hence disregarded. This number can be changed in line 72 if one's background noise is different, yet consistently so, from intensities of 5 and below.

Next follows how the report table is built up with its headers and which values should be placed. The program is done running when the report table appears.

The code

```
from collections import Counter
from ij import IJ
from ij import process
from ij.measure import ResultsTable

# get the current image as an ImagePlus
imp = IJ.getImage()

# convert ImagePlus to ColorProcessor
color_processor = process.ColorProcessor(imp.getBufferedImage())

# respect ROI
roi = imp.getRoi()
if roi: color_processor.setRoi(roi)

# get green values as bytes from channel
green_pixels = color_processor.crop().getChannel(2)
green_pixels = map(lambda p: p & 0xff, green_pixels)

red_pixels   = color_processor.crop().getChannel(1)
red_pixels = map(lambda p: p & 0xff, red_pixels)

# to avoid yellow (which shows up in red and green pixels)
# count only green pixels if equal to or more intense than red
minus_yellow = [x if x>=y else 0 for x,y in zip(green_pixels, red_pixels)]

green_pixels = minus_yellow
#print(green_pixels[:100])

### VISUAL TEST ###
#theight = 1200
#twidth = 1920
#mij = IJ.createImage("Worm", "8-bit black", twidth, theight, 1)
#mip = mij.getProcessor()
#a = []
# convert flat list to square (list of lists)
#for i in range(theight):
#    a.append(green_pixels[twidth*i:twidth*(i+1)])

# set each pixel individually in the new image
#for i in range(twidth):
#   for j in range(theight):
#      mip.putPixel(i, j, a[j][i])

#mij.show()

# for some reason imageJ bytes range -128 to 127

# convert signed java ints to unsigned ints by bitwise &, 0xff => 8bit, 0xff => 16bit ... so on
# wikipedia two's complement for more info
```

```
#gp = Counter(map(lambda p: p & 0xff, green_pixels))
# maybe clearer in binary, signed numbers use the first bit:
# -4 is 100, and 100 & 111 = 100, but in unsigned convention 4 is 100
# -3 is 101, becomes 5
# -2 is 110, becomes 6
# -1 is 111, becomes 7
#green_pixels = map(lambda p: p & 0b11111111, green_pixels)

# now use a python collection counter to get the number of times each pixel is observed
# counts = Counter(green_pixels)

# print if curious
# print(counts)

# but what we really want is:
# total intensity or pixel intensity * number of pixels
total = 0.0
ic = {x:0.0 for x in range(256)}
N = 0.0 # number of non-zero pixels
for pixel in green_pixels:
	# ignore 6> pixels
	if pixel < 6:
		continue
	# sum green intensity (i.e. value * number of pixels)
	total += pixel
	ic[pixel] += 1
	# sum number of pixels
	N += 1
#print(ic)
# report to a results table
#rt = ResultsTable()
rt = ResultsTable.getResultsTable()
# first set column headers
rt.setHeading(0, "Intensity")
rt.setHeading(1, "# Green")
rt.setHeading(2, "I/N")
rt.setHeading(3, "Name")
# then add values
rt.incrementCounter()
rt.addValue(0, total)
rt.addValue(1, N)
rt.addValue(2, total/float(N))
rt.addValue("Name", imp.getTitle())
rt.show("Green Intensity Scaled by the Number of Green Pixels")

# be careful with selections, they might drop out lots of pixels at 1
# and result in seemingly higher values...
```
