# Supplementary material for: Youthful and age‐related matreotypes predict drugs promoting longevity
Source: Aging Cell. 2021 Aug 4;20(9):e13441. doi: 10.1111/acel.13441 (PMC8441316; doi:10.1111/acel.13441)
Supplement: Supplementary file 15 — Supplementary Material [file ACEL-20-e13441-s007.pdf]

Extended Experimental Procedure

Aging matreotype definition (extended)

To define the human aging matreotype, we performed a literature search and extracted the age-association of all genes involved in forming the human matrisome. The inclusion criteria for the meta-analysis require whole genome coverage for each study, availability of the full dataset including negative data, and that the publication has undergone peer-review. We aggregated information stemming from multiple different data types ranging from age-dependent differential expression (Komljenovic et al. 2019; Yang et al. 2020), age-association (Dönertaş et al. 2018), age-correlation of expression (Yang et al. 2015), gene products directly linked to aging or linked to known aging regulators (Wang et al. 2009), manually curated age-related genes or regulating genes related to aging (Magalhães & Toussaint 2004), a mammalian meta-analysis of age-regulation (Magalhães et al. 2009), transcriptome biomarkers of healthy aging (Sood et al. 2015), age-associated DNA methylation (Bacalini et al. 2015; Bell et al. 2012). A large part of aging datasets were obtained from a large-scale meta-analysis conducted by (Blankenburg et al. 2018). If the datasets have not yet been subjected to a significance cutoff, we applied multiple testing corrected (Benjamini-Hochberg) threshold of 0.05 to each dataset if applicable. Tissue-specific differences were not further investigated due to confounding in the meta-analysis. Studies analyzing individual tissues were treated as separate sources. To define the aging matrisome, we

acquired data from at least three sources implicating the gene in the aging process. Studies that offer directionality were further utilized to determine matrisome genes that were upregulated or downregulated with age using the same thresholds.

Manual lifespan measurements

Manual scoring of lifespan as previously described by Ewald *et. al.* 2016 (Ewald et al. 2016). In brief, about 100 day-2 adult *C. elegans* were picked to NGM plates containing the solvent either water or 0.1% dimethyl sulfoxide (DMSO) alone as control or tretinoin (Sigma PHR1187), hyaluronic acid (Sigma H5388), chondroitin sulfate (Sigma 27042). Vitamin B12 (Sigma, PHR1234), chrysin (Santa Cruz Biotech, sc-204686) and dapsone (Santa Cruz Biotech, sc-203023A) lifespans were performed in 1.25% DMSO and 50 μM FUdR in 24 well-plates, 4 wells per condition and 30 worms per well, transferred at L4 developmental stage. Animals were classified as dead if they failed to respond to prodding. Exploded, bagged, burrowed, or animals that left the agar were excluded from the statistics. The estimates of survival functions were calculated using the product-limit (Kaplan-Meier) method. The log-rank (Mantel-Cox) method was used to test the null hypothesis and calculate *P* values (JMP software v.14.1.0.).

Automated survival assays using the lifespan machine

Automated survival analysis was conducted using the lifespan machine described by Stroustrup and colleagues (Stroustrup et al. 2013). Approximately 500 L4 animals were

resuspended in M9 and transferred to NGM plates containing 50 µM 5-Fluoro-2'deoxyuridine (FUdR) seeded either with OP50 bacteria, or with RNAi bacteria supplemented with 100 µg/ml carbenicillin, or with heat-killed OP50 bacteria, or with UV-inactivated *E. coli* strain NEC937 B (OP50 uvrA; KanR) containing 100 µg/ml carbenicillin. Animals were kept at 20°C until measurement. Tight-fitting Petri dishes (BD Falcon Petri Dishes, 50 x 9 mm) were used for lifespan experiments. Tight-fitting plates were dried without lids in a laminar flow hood for 40 minutes before starting the experiment. Air-cooled Epson V800 scanners were utilized for all experiments operating at a scanning frequency of one scan per 10 – 30 minutes. Temperature probes (Thermoworks, Utah, U.S.) were used to monitor the temperature on the scanner flatbed and maintain 20°C. Animals that left the imaging area during the experiment were censored. Population survival was determined using the statistical software R (Ihaka & Gentleman 2012) with the survival (Therneau & Grambsch 2000) and survminer (https://rpkgs.datanovia.com/survminer/) packages. Lifespans were calculated from the L4 stage (= day 0).

Compound preparation for lifespan and oxidative stress assays

Compounds are received freeze-dried, except for the liposomes, which were acquired in 100% saturated suspension. All compounds were blinded with a serial number. Compounds and control solvents are administered to *C. elegans* by mixing it in the Nematode Growth Medium (NGM) immediately before pouring the plates. Compound

stock solutions were made by dissolving 100 mg/mL in their solvent, 100% DMSO for genistein, water for the royal jelly oil in lecithin-based nanoemulsion. The royal jelly oil was prepared from dispersing royal jelly powder in oil (mygliol) but it was not soluble in the NGN agar *C. elegans* culturing plates. In a second step, we encapsulated the royal jelly oil by homogenizing the oil with lecithin. The liposomal genistein and empty liposomes were suspended in water. These stocks were consequently used to make dilution series. The final concentration of DMSO on the lifespan plates did not exceed 0.2%. The *C. elegans* strain TJ1060 was age-synchronized by extracting the eggs with bleach and were made infertile by culturing at 25°C from egg to day-1 of adulthood. On day-2-of adulthood, 30-40 animals were placed per 6 cm plates, four plates for each compound. Subsequently, the plates are loaded onto the scanners, kept in a controlled environment at 20°C. Every scanner includes at least four control plates. For the manual lifespan at 25°C, three plates were used per compound, and death events were counted once per day.

Oxidative stress assays

Oxidative stress assay was modified from Ewald *et al.,* 2017 (Ewald et al. 2017). *C. elegans* of the L1 or day-1-adult stage were shifted on compound-containing or control plates washed off at the indicated time point, incubated with 14 mM sodium arsenite (Honeywell International 35000) in U-Shaped 96 well plates, and put into the wMicroTracker (MTK100) for movement scoring. For statistical analysis, the area under

the curve was measured, and the mean for each run was calculated. Statistical analysis was performed by using a paired sample *t*-test.

Quantifying total collagen over protein content.

Collagen levels were determined by hydroxyproline content as described in Teuscher *et al.*, 2019 (Teuscher et al. 2019). In brief, about 10 000 TJ1060 *C. elegans* eggs were placed at 25°C until day-1 of adulthood and then transferred on plates containing the compounds at 20°C. Day-8-adult animals were harvested for the collagen and protein assays.

References:

Bacalini MG, Boattini A, Gentilini D, Giampieri E, Pirazzini C, Giuliani C, Fontanesi E, Remondini D, Capri M, Rio AD, Luiselli D, Vitale G, Mari D, Castellani G, Blasio AMD, Salvioli S, Franceschi C & Garagnani P (2015) A meta-analysis on age-associated changes in blood DNA methylation: results from an original analysis pipeline for Infinium 450k data. *Aging* 7, 97–109.
Bell JT, Tsai P-C, Yang T-P, Pidsley R, Nisbet J, Glass D, Mangino M, Zhai G, Zhang F, Valdes A, Shin S-Y, Dempster EL, Murray RM, Grundberg E, Hedman AK, Nica A, Small KS, Consortium TM, Dermitzakis ET, McCarthy MI, Mill J, Spector TD & Deloukas P (2012) Epigenome-Wide Scans Identify Differentially Methylated Regions for Age and Age-Related Phenotypes in a Healthy Ageing Population. *Plos Genet* 8, e1002629.
Blankenburg H, Pramstaller PP & Domingues FS (2018) A network-based meta-analysis for characterizing the genetic landscape of human aging. *Biogerontology* 19, 81–94.
Dönertaş HM, Valenzuela MF, Partridge L & Thornton JM (2018) Gene expression-based drug repurposing to target aging. *Aging Cell* 17, e12819.
Ewald CY, Hourihan JM & Blackwell TK (2017) Oxidative Stress Assays (arsenite and tBHP) in Caenorhabditis elegans. *BIO-PROTOCOL* 7.
Ewald CY, Marfil V & Li C (2016) Alzheimer-related protein APL-1 modulates lifespan

through heterochronic gene regulation in Caenorhabditis elegans. *Aging cell*, 1–12.
Ihaka R & Gentleman R (2012) R: A Language for Data Analysis and Graphics. *J Comput Graph Stat* 5, 299–314.
Komljenovic A, Li H, Sorrentino V, Kutalik Z, Auwerx J & Robinson-Rechavi M (2019) Cross-species functional modules link proteostasis to human normal aging R. Guigó, ed. *Plos Comput Biol* 15, e1007162.
Magalhães JP de, Curado J & Church GM (2009) Meta-analysis of age-related gene expression profiles identifies common signatures of aging. *Bioinform Oxf Engl* 25, 875–81.
Magalhães JP de & Toussaint O (2004) GenAge: a genomic and proteomic network map of human ageing. *Febs Lett* 571, 243–7.
Sood S, Gallagher IJ, Lunnon K, Rullman E, Keohane A, Crossland H, Phillips BE, Cederholm T, Jensen T, Loon LJC van, Lannfelt L, Kraus WE, Atherton PJ, Howard R, Gustafsson T, Hodges A & Timmons JA (2015) A novel multi-tissue RNA diagnostic of healthy ageing relates to cognitive health status. *Genome Biol* 16, 185.
Stroustrup N, Ulmschneider BE, Nash ZM, López-Moyado IF, Apfeld J & Fontana W (2013) The Caenorhabditis elegans Lifespan Machine. *Nature Methods* 10, 665–670.
Teuscher AC, Statzer C, Pantasis S, Bordoli MR & Ewald CY (2019) Assessing Collagen Deposition During Aging in Mammalian Tissue and in Caenorhabditis elegans. *Methods Mol Biology Clifton N J* 1944, 169–188.
Therneau TM & Grambsch PM (2000) Modeling Survival Data: Extending the Cox Model. , 1–6.
Wang X, Zhao Y, Wong K, Ehlers P, Kohara Y, Jones SJ, Marra MA, Holt RA, Moerman DG & Hansen D (2009) Identification of genes expressed in the hermaphrodite germ line of C. elegans using SAGE. *BMC Genomics* 10, 213.
Yang J, Huang T, Petralia F, Long Q, Zhang B, Argmann C, Zhao Y, Mobbs CV, Schadt EE, Zhu J, Tu Z, Ardlie KG, Deluca DS, Segrè AV, Sullivan TJ, Young TR, Gelfand ET, Trowbridge CA, Maller JB, Tukiainen T, Lek M, Ward LD, Kheradpour P, Iriarte B, Meng Y, Palmer CD, Winckler W, Hirschhorn J, Kellis M, MacArthur DG, Getz G, Shablin AA, Li G, Zhou Y-H, Nobel AB, Rusyn I, Wright FA, Lappalainen T, Ferreira PG, Ongen H, Rivas MA, Battle A, Mostafavi S, Monlong J, Sammeth M, Mele M, Reverter F, Goldmann J, Koller D, Guigo R, McCarthy MI, Dermitzakis ET, Gamazon ER, Konkashbaev A, Nicolae DL, Cox NJ, Flutre T, Wen X, Stephens M, Pritchard JK, Lin L, Liu J, Brown A, Mestichelli B, Tidwell D, Lo E, Salvatore M, Shad S, Thomas JA, Lonsdale JT, Choi C, Karasik E, Ramsey K, Moser MT, Foster BA, Gillard BM, Syron J, Fleming J, Magazine H, Hasz R, Walters GD, Bridge JP, Miklos M, Sullivan S, Barker LK, Traino H, Mosavel M, Siminoff LA, Valley DR, Rohrer DC, Jewel S, Branton P, Sobin

LH, Qi L, Hariharan P, Wu S, Tabor D, Shive C, Smith AM, Buia SA, Undale AH, Robinson KL, Roche N, Valentino KM, Britton A, Burges R, Bradbury D, Hambright KW, Seleski J, Korzeniewski GE, Erickson K, Marcus Y, Tejada J, Taherian M, Lu C, Robles BE, Basile M, Mash DC, Volpi S, Struewing J, Temple GF, Boyer J, Colantuoni D, Little R, Koester S, Carithers NLJ, Moore HM, Guan P, Compton C, Sawyer SJ, Demchok JP, Vaught JB, Rabiner CA & Lockhart NC (2015) Synchronized age-related gene expression changes across multiple tissues in human and the link to complex diseases. *Sci Rep-uk* 5, 15145.
Yang J, Peng S, Zhang B, Houten S, Schadt E, Zhu J, Suh Y & Tu Z (2020) Human geroprotector discovery by targeting the converging subnetworks of aging and age-related diseases. *Geroscience* 42, 353–372.
