# Supplementary material for: Youthful and age‐related matreotypes predict drugs promoting longevity
Source: Aging Cell. 2021 Aug 4;20(9):e13441. doi: 10.1111/acel.13441 (PMC8441316; doi:10.1111/acel.13441)
Supplement: Supplementary file 7 — Figure S7 [file ACEL-20-e13441-s008.pdf]

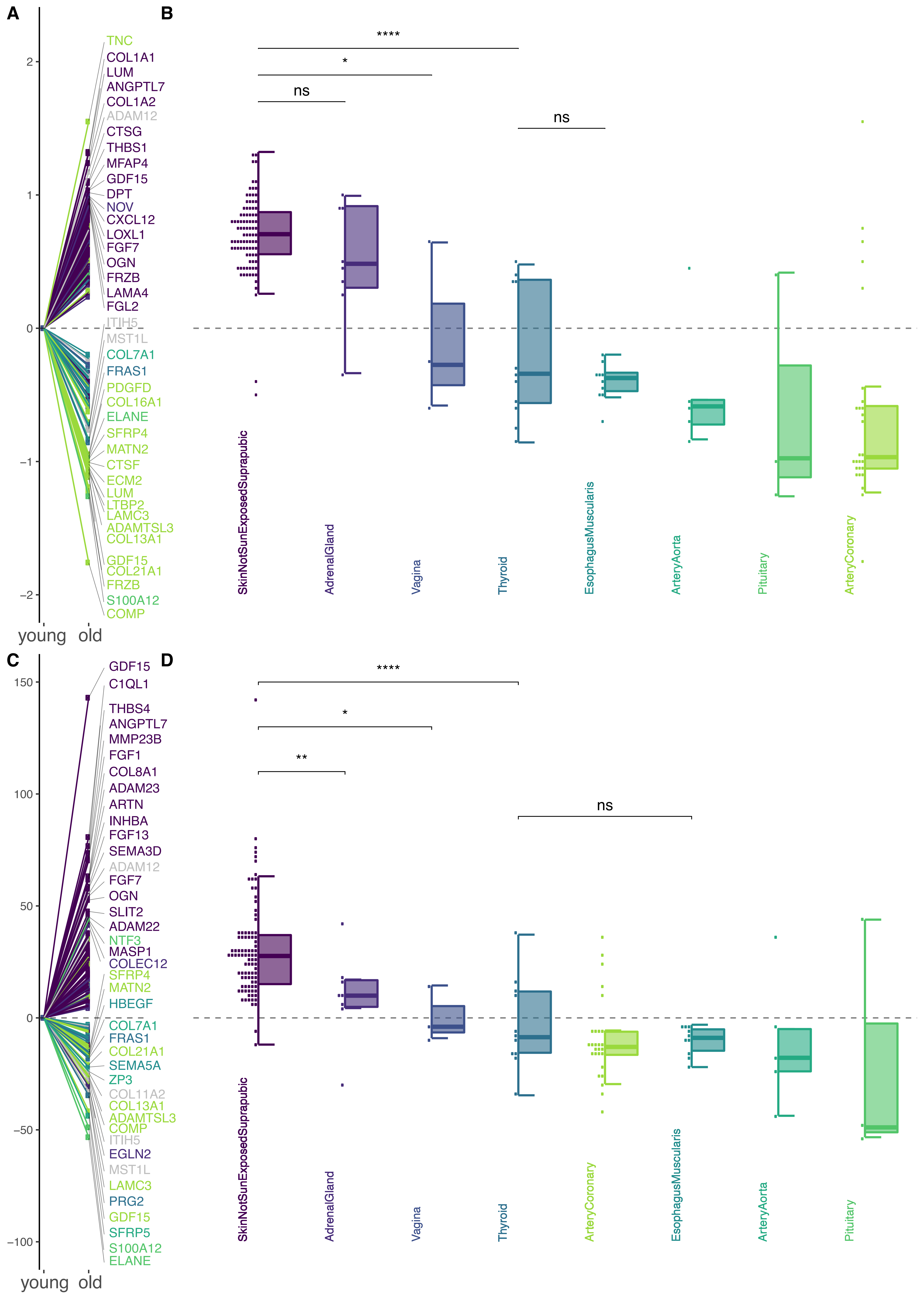

A
B
C
D
TNC
COL1A1
LUM
ANGPTL7
COL1A2
ADAM12
CTSG
THBS1
MFAP4
GDF15
DPT
NOV
CXCL12
LOXL1
FGF7
OGN
FRZB
LAMA4
FGL2
ITIH5
MST1L
COL7A1
FRAS1
PDGFD
COL16A1
ELANE
SFRP4
MATN2
CTSF
ECM2
LUM
LTBP2
LAMC3
ADAMTSL3
COL13A1
GDF15
COL21A1
FRZB
S100A12
COMP
young
old
****
*
ns
ns
SkinNotSunExposedSuprapubic
AdrenalGland
Vagina
Thyroid
EsophagusMuscularis
ArteryAorta
Pituitary
ArteryCoronary
GDF15
C1QL1
THBS4
ANGPTL7
MMP23B
FGF1
COL8A1
ADAM23
ARTN
INHBA
FGF13
SEMA3D
ADAM12
FGF7
OGN
SLIT2
ADAM22
NTF3
MASP1
COLEC12
SFRP4
MATN2
HBEGF
COL7A1
FRAS1
COL21A1
SEMA5A
ZP3
COL11A2
COL13A1
ADAMTSL3
COMP
ITIH5
EGLN2
MST1L
LAMC3
PRG2
GDF15
SFRP5
S100A12
ELANE
young
old
****
*
**
ns
SkinNotSunExposedSuprapubic
AdrenalGland
Vagina
Thyroid
ArteryCoronary
EsophagusMuscularis
ArteryAorta
Pituitary
